# Genetic enhancement of okra [*Abelmoschus esculentus* (L.) Moench] germplasm through wide hybridization

**DOI:** 10.3389/fpls.2023.1284070

**Published:** 2023-11-03

**Authors:** A. Suma, K. Joseph John, Kangila Venkataramana Bhat, Madhavan Latha, Chakkamadathil Jayasundaran Lakshmi, Mottaiyan Pitchaimuthu, V. A. M. Nissar, Polavakkalipalayam Palanisamy Thirumalaisamy, Chitra Devi Pandey, Sushil Pandey, Ashok Kumar, Raj Kumar Gautam, Gyanendra Pratap Singh

**Affiliations:** ^1^ ICAR-National Bureau of Plant Genetic Resources, Regional Station, Thrissur, Kerala, India; ^2^ ICAR-National Bureau of Plant Genetic Resources, New Delhi, India; ^3^ ICAR-Indian Institute of Horticultural Research, Bengaluru, Karnataka, India

**Keywords:** pre-breeding, crop wild relatives, amphidiploids, polyploidization, morphological characterization, colchicine

## Abstract

**Introduction:**

The introgression of genetic material from one species to another through wide hybridization and repeated back-crossing, plays an important role in genetic modification and enriching the cultivated gene-pool with novel genetic variations. Okra (*Abelmoschus esculentus* [(L.) Moench)] is a popular vegetable crop with high dietary fibre and protein, rich in essential amino acids, lysine and tryptophan. The wild *Abelmoschus* genepool has many desirable traits like ornamental value, short internodal length, more number of productive branches, extended bearing, perennation tendency, reduced fruit length (more consumer preferred trait), high mucilage content (medicinal value), abiotic stress tolerances such as drought, high temperature and biotic stress resistances such as okra Yellow Vein Mosaic Virus (YVMV) and Enation Leaf Curl Virus (ELCV) diseases. The repeated use of elite breeding lines led to narrowing of the genetic base of the okra crop, one of the major factors attributed to breakdown of resistance/ tolerance to biotic stresses. YVMV and ELCV are the two major diseases, causing significant yield loss in okra. Hence, wide hybridization was attempted to transfer tolerance genes from wild species to the cultivated genepool to widen the genetic base.

**Material and methods:**

The screening of germplasm of wild *Abelmoschus* species at hotspots led to the identification of tolerant species (*Abelmoschus pungens var. mizoramensis*, *A. enbeepeegeearensis*, *A. caillei*, *A. tetraphyllus* and *A. angulosus var. grandiflorus*), which were further used in a wide-hybridization programme to generate interspecific hybrids with the cultivated okra. Presence of pre- and post-zygotic barriers to interspecific geneflow, differences in ploidy levels and genotype specific variations in chromosome numbers led to varying degrees of sterility in F_1_ plants of interspecific crosses. This was overcome by doubling the chromosome number of interspecific hybrids by applying Colchicine at the seedling stage. The 113 cross derivatives generated comprising amphidiploids in the F_1_ generation (30), F_3_ (14), one each in F_2_ and F_4_ generations, back cross generation in BC_1_F_2_ (03), BC_1_F_3_ (25), and BC2F3 (02), crosses between amphidiploids (27), multi-cross combinations (07) and inter-specific cross (between *A. sagittifolius* × *A. moschatus* subsp. *moschatus*) selfed derivatives at F_8_ generation (03) were characterized in the present study. Besides they were advanced through selfing and backcrossing.

**Results and Discussion:**

The amphidiploids were found to possess many desirable genes with a considerable magnitude of linkage drag. Majority of the wide cross derivatives had an intermediate fruit morphology and dominance of wild characters *viz*., hispid fruits, stem, leaves, tough fruit fibre, vigorous perennial growth habit and prolonged flowering and fruiting. The fruit morphology of three BC progenies exhibited a high morphological resemblance to the cultivated okra, confirming successful transfer of useful genes to the cultivated okra genepool. The detailed morphological characteristics of the various combinations of *Abelmoschus* amphidiploids and the genetic enhancement of the genepool achieved in this process is reported here.

## Introduction

Introgression, the transfer of genes between species mediated primarily by backcrossing (Anderson, 1949; Twyford and Ennos, 2012) plays an important role in the evolution of plant species. It results in genetic modification and enrichment of the genepool for crop improvement programs. The extent of introgression in nature is limited though not prevented by the existence of reproductive barriers that filter natural geneflow (Mallet, 2005; Slotte et al., 2008). Several successful attempts to break interspecific barriers to gene flow have been documented in different crops, which resulted in new genomic combinations (Joseph et al., 2017). Though breeders are consistently interested in introgressing genes conferring desirable traits from wild to cultivated species, the process is elaborate due to the predominance of linkage drag exhibited by the introgressed lines.

Okra (*Abelmoschus esculentus* L. Moench), a popular traditional vegetable under the family Malvaceae, is grown throughout the tropical and subtropical regions of the world and in warmer parts of the temperate zone. Tender fruits are used as vegetables, in addition to their leaves, buds, and flowers being consumed especially in West Africa (Sharma, 1993). Further, the fiber extracted from the waste stems can be used to make paper pulp or fuel ( Gogoi et al., 2017), while the foliage can be used as biomass (Sharma, 1993). Okra mucilage has potential use in food and non-food products and for medicinal purposes (Ortaç et al., 2018; Peng et al., 2019). Studies also demonstrated the presence of bioactive compounds in plant parts such as leaves, flowers, and seeds (Adelakun et al., 2009; Zhang et al., 2020; Adetuyi et al., 2012; Olawuyi and Lee, 2021). Recently, the effects of okra leaves, fruits, and seed extracts on European sea bass (*Dicentrarchus labrax*) leukocytes and their cytotoxic, bactericidal, and antioxidant properties were reported by Guebebia et al. (2023). Okra has a vast potential for earning foreign exchange, as it has a significant share in fresh vegetable export of India (2,442.96 million US$ fresh vegetable export during 2022–2023; https://agriexchange.apeda.gov.in/IndExp/PortNew.aspx accessed on Aug 3, 2023).

Breeding efforts are in full swing to develop novel ideotypes with branching behavior, shorter internodes, multi-flory, ramiflory, and nutritional quality. However, the cultivation of okra is facing serious threats due to two major viral diseases, namely, okra Yellow Vein Mosaic Virus (YVMV) and Enation Leaf Curl Virus (ELCV) diseases. Among them, YVMV disease is the most severe constraint responsible for 90% to 100% yield loss depending upon the stage of infection. These diseases reduce the yield substantially and affect the marketability of the fruits (Sanwal et al., 2014a). The virus is readily transmitted by grafting and through the insect vector, whitefly (*Bemisia tabaci* Gen.) (Varma, 1952). Host-plant tolerance to viruses is one of the most practical, economical, and environment-friendly strategies for reducing yield loss in okra (Ayam et al., 2018). Intensive efforts were underway in India to develop virus-tolerant varieties, and it resulted in the release of several varieties, namely, Pusa Sawani, Arka Anamika, Arka Abhay, and Parbhani Kranti. The host-plant tolerance has been overcome by the evolution of new variants of the viruses because of the development of new strains or due to recombination in virus strains (Sanwal et al., 2014b). Another major reason for the breakdown of resistance would be due to the emergence of the polyphagous ‘B’ biotype of *B. tabaci*, with its increased host range resulting in the infection of the Gemini virus in previously unaffected crops (Chowda-Reddy et al., 2012). Presently, none of the cultivated okra varieties or hybrids have stable tolerance to YVMV disease (Sandeep et al., 2022).

Undomesticated crop wild relatives are rich sources of genes providing resistance to various diseases, pests, and unfavorable environmental conditions (Rattan and Kumar, 2020). In addition to disease resistance, other agronomic traits such as male sterility (Sigareva and Earle, 1997 in cabbage) and abiotic stress tolerance (Wei et al., 2015 in tobacco) have also been transferred by wide hybridization. Successful development and characterization of amphidiploids have been reported in several crops such as wheat (Zhang et al., 2016; Song et al., 2019; Klimushina et al., 2020; Zuo et al., 2020; Ren et al., 2022), rice (Wang et al., 2013; Kumar et al., 2019), peanut (de Paula et al., 2017; Li-Na et al., 2017; Favero et al., 2020; Ballén-Taborda et al., 2022), Brassica (Thomsen, 1990; Song et al., 1993), Vigna (Mjnocha et al., 1991), Dianthus (Nimura et al., 2006), Cucumis (Chen et al., 2002), and Solanum (Khan et al., 2013; Zhou et al., 2018). Even though many workers have attempted and succeeded in developing interspecific hybrids, the introgressed lines could be utilized only after eliminating the fertility barriers associated with the hybrids.

Dark green fruits, high mucilage content, extended bearing, perennation propensity, high branching, reduced fruit length, and tolerance to drought, high temperature, and okra YVMV disease are some of the favorable characteristics of the wild okra species (Sandeep et al., 2022). John et al. (2013a) reported that wild relatives, namely, *Abelmoschus angulosus* var. *grandiflorus* Thwaites, *Abelmoschus crinitus* Wall., *Abelmoschus ficulneus* (L.) Wight & Arn., *Abelmoschus tetraphyllus* (Roxb. ex Hornem.) Wall., *Abelmoschus pungens* var. *mizoramensis* K.J.John, Krishnaraj & K.Pradheep, and *Abelmoschus enbeepeegearensis* K.J.John, Scariah, Nissar, K.V.Bhat & S.R.Yadav did not express any YVMV disease symptoms under field epiphytotic conditions. Similarly, Gangopadhyay et al. (2016) reported field-level tolerance to this disease in accessions belonging to *Abelmoschus caillei* (A.Chev.) J.M.C.Stevels, *Abelmoschus manihot* (L.) Medik., and *Abelmoschus moschatus* Medik. species. Further, *A. manihot* (L.) Medik. subsp. *tetraphyllus* (Roxb. Ex Hornem.) Borss. Waalk. was reported as one of the most prominent species exploited worldwide as a source of resistance/tolerance to YVMV, jassids, and fruit borer (Gangopadhyay et al., 2016; Badiger and Yadav, 2019; Patel et al., 2021). Santhiya et al. (2022) also reported the promising accessions of *A. moschatus* (IC141055), *A. tetraphyllus* (IC90476-1), and *A. caillei* (Sikkim), which showed very low incidence of YVMV disease and no incidence of ELCV disease under natural epiphytotic screening. Accessions IC203833 and IC470751 of *A. angulosus* were reported by Singh et al. (2023a) to be highly resistant to YVMV disease under artificial screening conditions using viruliferous whitefly-mediated mass inoculation. However, elaborate and systematic studies on screening of available wild relatives of *Abelmoschus* and their utilization in breeding programs are still limited.

Hence, an intensive and systematic wide hybridization or pre-breeding program is required to identify and transfer the genes for tolerance to abiotic and biotic stresses from the wild *Abelmoschus* species to the cultivated species. Several earlier attempts of wide hybridization in okra have been reported, which include Pal et al. (1952) and Kuwada (1966) for *A. esculentus* with *Abelmoschus tuberculatus*; Kuwada (1974) for *A. tuberculatus* with *A. manihot*; Akhond et al. (2000) for *A. esculentus* with *A. moschatus*; and Samarajeewa (2003) for *A. esculentus* with *A. angulosus* and *A. esculentus* with *A. caillei*. Further, Manju and Gopimony (2009) succeeded in the development of the variety ‘Anjitha’ by inducing mutation through gamma irradiation of interspecific hybrids of *A. esculentus* cv. *Kiran* × *A. manihot*. Further, Nagaraju et al. (2019) carried out interspecific hybridization using 10 wild species and Amiteye et al. (2019) using *A. esculentus* and *A. caillei*. Rattan and Kumar (2020) developed the protocol for embryo rescue of interspecific hybrids involving *A. esculentus* × *A. tetraphyllus*. Badiger et al. (2023) elaborated the pollen germination, pollen–pistil interaction, and crossability studies in interspecific and induced colchiploid populations involving crosses between *A. esculentus* and wild species, viz., *A. manihot* var. *tetraphyllus* and *A. moschatus*. Even though several studies on wide hybridization and development of successful amphidiploids are available, this is the first-of-its-kind report on the characterization of amphidiploids developed through wide hybridizations in the *Abelmoschus* genus. The present report addresses the following objectives: 1) to document the morphological variability in amphidiploid derivatives among different wild *Abelmoschus* species and okra and 2) to identify the utility of the *Abelmoschus* amphidiploids as genetic stocks and sources of useful genes in a variety of development programs.

## Materials and methods

Crossability studies and wide hybridization work were started in 2009–2013 and 2015–2017 under the Indian Council of Agricultural Research (ICAR)—National Agricultural Innovation Project (NAIP) and continued under the ICAR-Extramural Project of the ICAR-Horticultural Sciences Division and later under the ICAR-Emeritus Scientist Programme (2018–2021). Initially, under the NAIP, interspecific hybridization was attempted between different *Abelmoschus* species, primarily for establishing crossability relationships, thereby understanding the genetic relationships between the species based on fertility and secondarily to derive new recombinants to be used in breeding programs (John et al., 2013a). Further, under the subsequent project, the available wild species (44 accns. of 11 *Abelmoschus* taxa) conserved in the Medium Term Storage (MTS) module of ICAR—National Bureau of Plant Genetic Resources (NBPGR), Regional Station, Thrissur, Kerala, India, were evaluated for resistance/tolerance to okra YVMV and ELCV diseases at three hot spot locations, namely, Varanasi, Guntur, and New Delhi, India. The wild *Abelmoschus* taxa include *A. moschatus* subsp. *moschatus*, *A. caillei*, *A. tetraphyllus*, *A. pungens* var. *mizoramensis*, *A. angulosus* var. *grandiflorus*, *A. enbeepeegearensis*, *A. crinitus*, *Abelmoschus palianus*, *A. tuberculatus*, *A. ficulneus*, and *Abelmoschus sagittifolius*. This led to the identification of tolerant accessions of *A. enbeepeegearensis* and *A. angulosus* var. *grandiflorus*. These tolerant accessions along with other reported sources of virus tolerance (John et al., 2013a) were selected for wide hybridization. It includes 18 accessions of *A. esculentus* (10 accns. and eight released varieties), one of *A. caillei*, and eight of wild *Abelmoschus* spp., the details of which are given in **Table 1**.

**Table 1:** The details of cultivated and wild *Abelmoschus* germplasm used in the wide hybridization program.

S. no.	Accession no.	Name of the species	Status	Collection source
1	Pusa Sawani	*Abelmoschus esculentus*	Cultivated	Released variety
2	Arka Anamika	*A. esculentus*	Cultivated	Released variety
3	Parbhani Kranti	*A. esculentus*	Cultivated	Released variety
4	Salkeerthi	*A. esculentus*	Cultivated	Released variety
5	Kashi Lalima	*A. esculentus*	Cultivated	Released variety
6	Kashi Vibhuti	*A. esculentus*	Cultivated	Released variety
7	Hissar Unnat	*A. esculentus*	Cultivated	Released variety
8	South Canara Local	*A. esculentus*	Cultivated	Karnataka, India
9	IC412987	*A. esculentus*	Cultivated	Tamil Nadu, India
10	IC260106	*A. esculentus*	Cultivated	Tamil Nadu, India
11	EC306741	*A. esculentus*	Cultivated	Singapore
12	IC22232	*A. esculentus*	Cultivated	Madhya Pradesh, India
13	IC31340C	*A. esculentus*	Cultivated	India
14	IC31398A	*A. esculentus*	Cultivated	India
15	IC32398A	*A. esculentus*	Cultivated	India
16	IC265650	*A. esculentus*	Cultivated	Kerala, India
17	EC169416	*A. esculentus*	Cultivated	–
18	EC169415	*A. esculentus*	Cultivated	Philippines
19	IC566817	*Abelmoschus caillei*	Cultivated	India
20	IC624222	*Abelmoschus pungens* var. *mizoramensis*	Wild	Mizoram, India
21	IC624235	*A. pungens* var. *mizoramensis*	Wild	Nagaland, India
22	IC624236	*A. pungens* var. *mizoramensis*	Wild	Nagaland, India
23	IC253122	*Abelmoschus tetraphyllus*	Wild	India
24	IC613527	*Abelmoschus angulosus* var. *grandiflorus*	Wild	Kerala, India
25	IC582757	*Abelmoschus enbeepeegearensis*	Wild	Kerala, India
26	IC624218	*Abelmoschus palianus*	Wild	Chhattisgarh, India
27	EC306750	*Abelmoschus moschatus* (variant)	Wild	Singapore
28	IC624232	*A. moschatus* subsp. *moschatus*	Wild	Kerala, India
29	IC470750	*Abelmoschus sagittifolius*	Wild	Kerala, India

### Hybridization

Hybridization is the critical step for obtaining successful hybrids, as anthesis time, anther dehiscence, and pollen viability of the wild species affect the success of the process. Mature flower buds of the female parent were emasculated before anthesis by shaving off the anthers in the previous evening and covered with butter paper selfing bags. Ready-to-open flower buds of the male parent were also covered at the same time to prevent pollen contamination. Pollen from the male parent was collected and dusted onto the receptive stigma of emasculated flowers between 9:00 AM and 10:00 AM (next day) under Thrissur, Kerala conditions and again covered with butter paper bags to avoid pollination with undesirable pollen and then tagged. The selfing bags were removed 3 days after pollination to allow the fruits to develop properly. The tagged fruits upon maturity were harvested, and hybrid seeds were extracted and stored for 3–6 months in a cool place.

### Colchicine treatment

**Figure 1:**
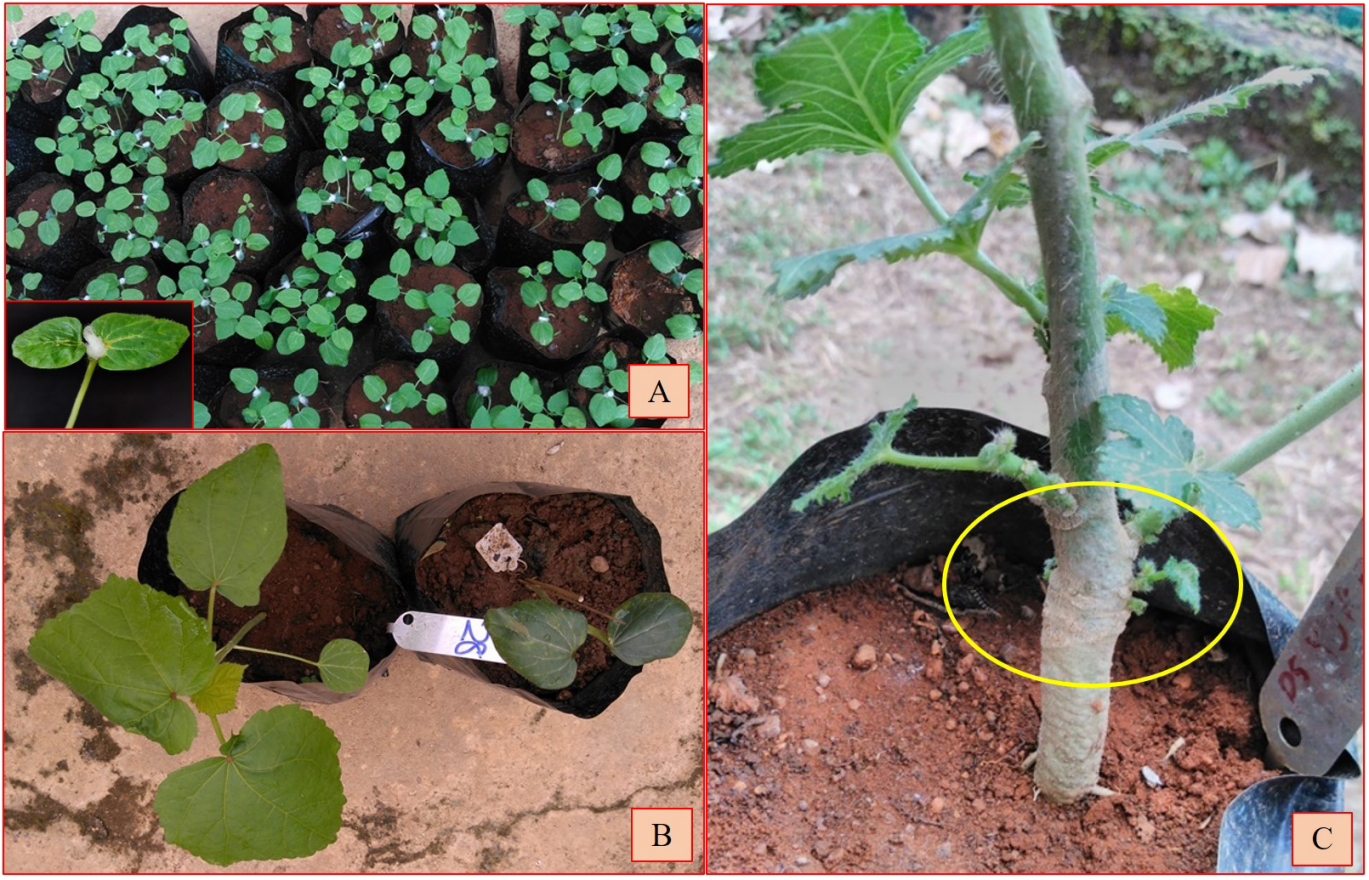
**(A)** Colchicine treatment using cotton swab method on interspecific hybrid seedlings. Inset: close-up photograph of colchicine treatment. **(B)** Colchicine un-treated and treated seedlings. **(C)** Bulging at the colchicine-treated site.

The interspecific F_1_ seeds were scarified by rubbing on sandpaper and soaked overnight in distilled water before sowing. Seedlings were raised in polybags, and half the number of seedlings in all cross combinations were subjected to colchicine treatment. The untreated seedlings were transplanted into pots and allowed to grow under optimal conditions. The untreated seedlings were grown separately, and it was found that the F_1_ set fruits were without seed set. The pollen grains from these hybrids were found to be pale cream colored, indicating the sterility of the pollen. In order to overcome the sterility in the interspecific hybrids, emerging seedlings at the third leaf stage (epicotyl leaves fully grown and third leaf emergence stage) were subjected to colchicine treatment, 0.10% for 3 days from 7:00 AM to 11:00 AM at 20–30-min intervals using cotton swab method (**Figure 1A**). As per this method, the cotton swab placed on the apical meristem was soaked with 0.10% colchicine at regular intervals with one drop of the solution. After completion of the application each day, the cotton swab was washed with distilled water, and on the fourth day, the cotton swab was removed (Joseph et al., 2017). The treated plants were regularly sprayed with artificial growth formulations of mineral supplements to boost growth. All treated seedlings were provided with support to withstand weight due to enlarged tissue on account of chromosome doubling, and plants were given an optimal growth environment by shifting them to a mist house.

### General observations on colchicine-treated seedlings

**Figure 2:**
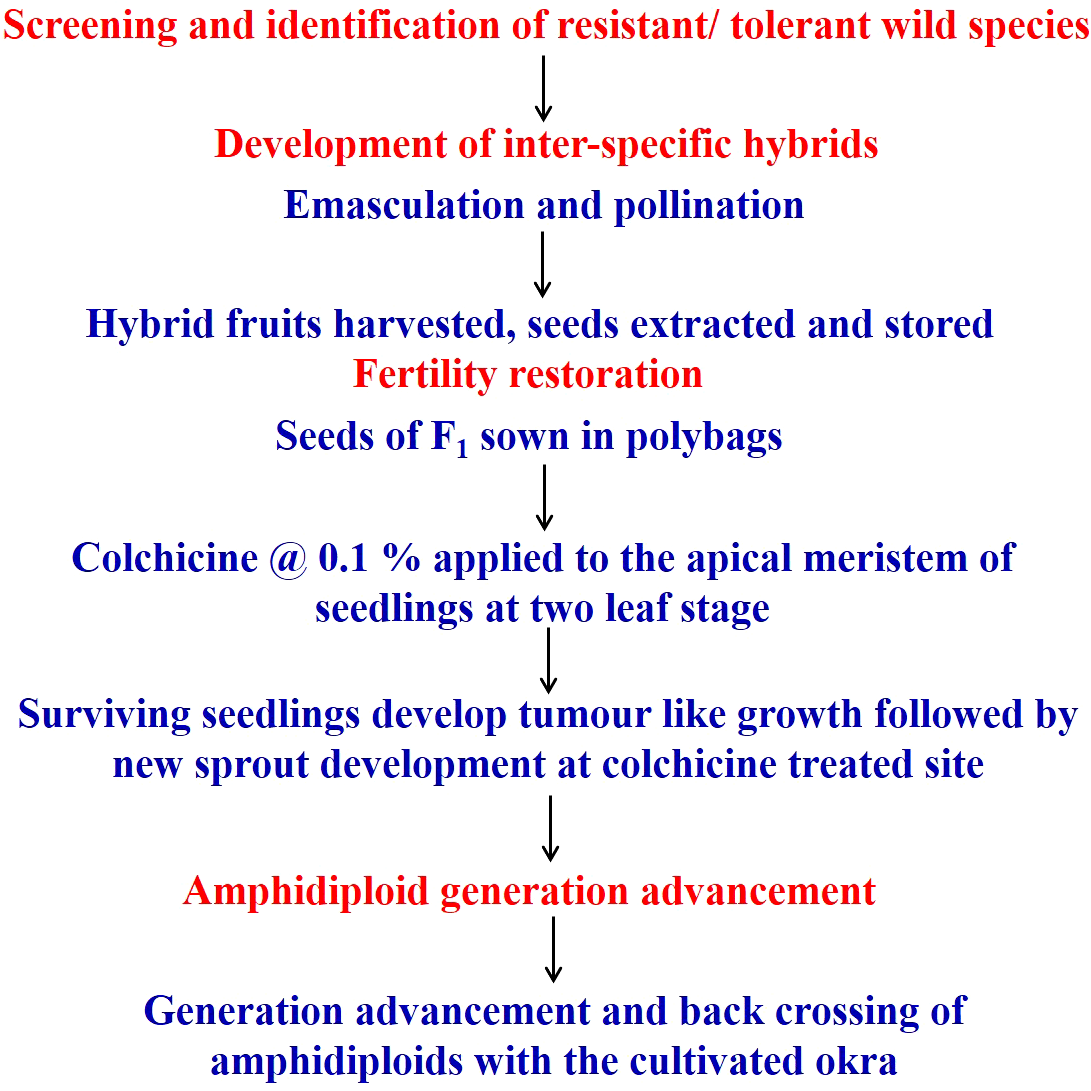
Flowchart of wide hybridization, *Abelmoschus* amphidiploid development, and generation advancement.

**Figure 3:**
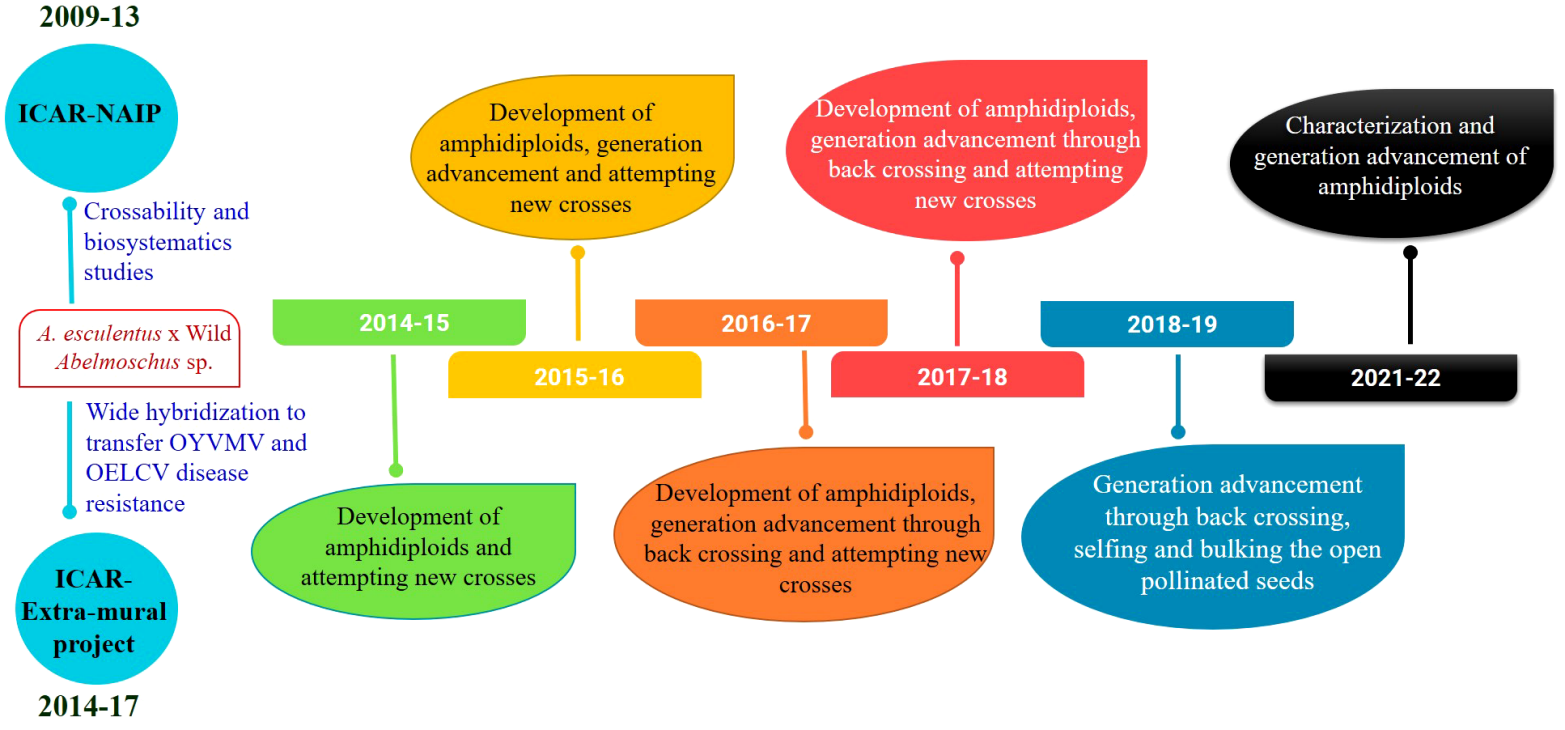
Timeline showing the wide hybridization program in okra.

The seedlings exhibited scorching symptoms in the apical region after the colchicine treatment, but mortality depended on the genotype of the seedlings and the prevailing environmental conditions. The third leaf was crumbled or retarded in its growth and in some cases was partially dried. The epicotyl leaves became succulent and turgid and changed their color from light green to dark green (**Figure 1B**). The epicotyl leaf petioles became shortened compared to the normal seedlings. It took nearly 3–4 weeks for the seedling to overcome the stress induced by the colchicine treatment. Bulging near the collar region, resembling a tumor-like appearance developed at the site of colchicine treatment, was considered an indication of polyploidization (Suma et al., 2020) (**Figure 1C**). The surviving seedlings were transplanted to standard pots and provided optimal growth conditions following a set of practices recommended for okra. The fertile amphidiploids were further selfed for one more generation to stabilize the genetic architecture (Joseph et al., 2017). Hence, the seeds from the amphidiploids (C_0_ generation) were harvested and used for generation advancement for the next season. The flowchart detailing the development and advancement of amphidiploids and the timeline depicting the wide hybridization program in okra are presented in **Figures 2**, **3**, respectively.

### Naming of amphidiploids

The amphidiploids were named considering their generation, indigenous collection/exotic collection (IC/EC) number of the cultivated species (*A. esculentus* or *A. caillei*), and the name of the species/subspecies/variety taxon of the wild *Abelmoschus* used for hybridization followed by the plant number of the colchiploid selected. For instance, C_3_/106/mizo30-4 is an amphidiploid in which the capital letter “C” denotes the colchicine-treated offsprings (synonymous to “F”, the filial generation used in general hybridization events) and the subscript “3” the generation of the amphidiploid followed by the last two/three digits of the number of *A. esculentus* (e.g., 106 of IC260106 in the above-mentioned amphidiploid). It is followed by the first four letters of the lowest taxon of the wild *Abelmoschus* (mizo of *A. pungens* var. *mizoramensis*) species used in the hybridization, followed by the plant numbers of the amphidiploid selected in the subsequent generations. However, in C_1_Pusamizo and C_1_Arkamosc (variant) derivatives, the lowest taxon name of wild *Abelmoschus* species was followed by the last three digits of the IC/EC number of the wild species used in hybridization, viz., C_1_Pusamizo235-02 and C_1_Arkamosc (variant) 750-01, followed by plant number selected. Further, for convenience, the selfed derivatives in the F_2_ and F_3_ generations were coded as AM series and the multi-cross combinations as B series (**Tables 2B, E, F**). The surviving amphidiploids were further advanced through selfing up to the C_2_/C_3_ generation. The advanced amphidiploids were screened for tolerance/resistance to okra YVMV and ELCV diseases under natural epiphytotic conditions in New Delhi, Guntur (Andhra Pradesh) and Thrissur (Kerala), India, during 2017–2018. The amphidiploid derivatives presumed to have acquired genes from cultivated okra (with low spininess and intermediate fruit length) and found to be tolerant to YVMV disease along with a profuse and extended bearing habit were selected for generation advancement by selfing or backcrossing under the ICAR-Emeritus Scientist Programme.

**Table 2A:** Details of pedigree information of amphidiploid derivatives in F_1_ generation.

S. no.	Female parent	Male parent	Amphidiploid name	Gn
1	Pusa Sawani	*Abelmoschus pungens* var. *mizoramensis* IC624236	C_1_Pusamizo236-29	F_1_
2	Pusa Sawani	*A. pungens* var. *mizoramensis* IC624236	C_1_Pusamizo236-26	F_1_
3	Pusa Sawani	*A. pungens* var. *mizoramensis* IC624236	C_1_Pusamizo236-25	F_1_
4	Pusa Sawani	*A. pungens* var. *mizoramensis* IC624236	C_1_Pusamizo236-24	F_1_
5	Pusa Sawani	*A. pungens* var. *mizoramensis* IC624236	C_1_Pusamizo236-22	F_1_
6	Pusa Sawani	*A. pungens* var. *mizoramensis* IC624236	C_1_Pusamizo236-21	F_1_
7	Pusa Sawani	*A. pungens* var. *mizoramensis* IC624236	C_1_Pusamizo236-18	F_1_
8	Pusa Sawani	*A. pungens* var. *mizoramensis* IC624236	C_1_Pusamizo236-16	F_1_
9	Pusa Sawani	*A. pungens* var. *mizoramensis* IC624236	C_1_Pusamizo236-15	F_1_
10	Pusa Sawani	*A. pungens* var. *mizoramensis* IC624236	C_1_Pusamizo236-20	F_1_
11	Pusa Sawani	*A. pungens* var. *mizoramensis* IC624236	C_1_Pusamizo236-27	F_1_
12	Pusa Sawani	*A. pungens* var. *mizoramensis* IC624236	C_1_Pusamizo236-05	F_1_
13	Pusa Sawani	*A. pungens* var. *mizoramensis* IC624236	C_1_Pusamizo236-06	F_1_
14	Pusa Sawani	*A. pungens* var. *mizoramensis* IC624236	C_1_Pusamizo236-07	F_1_
15	Pusa Sawani	*A. pungens* var. *mizoramensis* IC624236	C_1_Pusamizo236-11	F_1_
16	Pusa Sawani	*A. pungens* var. *mizoramensis* IC624236	C_1_Pusamizo236-12	F_1_
17	Pusa Sawani	*A. pungens* var. *mizoramensis* IC624236	C_1_Pusamizo236-14	F_1_
18	Pusa Sawani	*A. pungens* var. *mizoramensis* IC624236	C_1_Pusamizo236-02	F_1_
19	Pusa Sawani	*A. pungens* var. *mizoramensis* IC624236	C_1_Pusamizo236-30	F_1_
20	Pusa Sawani	*A. pungens* var. *mizoramensis* IC624236	C_1_Pusamizo236-04	F_1_
21	Pusa Sawani	*A. pungens* var. *mizoramensis* IC624235	C_1_Pusamizo235-09	F_1_
22	Pusa Sawani	*A. pungens* var. *mizoramensis* IC624235	C_1_Pusamizo235-04	F_1_
23	Pusa Sawani	*A. pungens* var. *mizoramensis* IC624235	C_1_Pusamizo235-03	F_1_
24	Pusa Sawani	*A. pungens* var. *mizoramensis* IC624235	C_1_Pusamizo235-02	F_1_
25	Pusa Sawani	*A. pungens* var. *mizoramensis* IC624235	C_1_Pusamizo235-01	F_1_
26	Pusa Sawani	*A. pungens* var. *mizoramensis* IC624235	C_1_Pusamizo235-06	F_1_
27	Arka Anamika	*Abelmoschus moschatus* variant EC306750 (1)	C_1_Arkamosc (variant)750-01	F_1_
28	Arka Anamika	*A. moschatus* variant EC306750 (2)	C_1_Arkamosc (variant)750-02	F_1_
29	Arka Anamika	*A. moschatus* variant EC306750 (4)	C_1_Arkamosc (variant)750-04	F_1_
30	Arka Anamika	*A. moschatus* variant EC306750 (7)	C_1_Arkamosc (variant)750-07	F_1_

Gn, generation.

**Table 2B:** Details of pedigree information of selfed amphidiploid derivatives in F2, F3, and F4 generations.

S. no.	Amphidiploid code	Female parent	Male parent	Amphidiploid name	Gn
1	AM-2	*Abelmoschus esculentus* IC265650	*Abelmoschus pungens* var. *mizoramensis* IC0624222	C_3_/50/mizo30	F_3_
2	AM-3	*A. esculentus* IC260106	*A. pungens* var. *mizoramensis* IC0624222	C_3_/106/mizo30	F_3_
3	AM-4	*A. esculentus* IC265650	*A. pungens* var. *mizoramensis* IC0624222	C_3_/50/mizo30	F_3_
4	AM-5	*A. esculentus* IC265650	*A. pungens* var. *mizoramensis* IC0624222	C_3_/50/mizo30	F_3_
5	AM-6	*A. esculentus* IC265650	*A. pungens* var. *mizoramensis* IC0624222	C_3_/50/mizo24	F_3_
6	AM-8	*A. esculentus* IC265650	*A. pungens* var. *mizoramensis* IC0624222	C_3_/50/mizo30	F_3_
7	AM-9	*A. esculentus* IC260106	*A. pungens* var. *mizoramensis* IC0624222	C_3_/106/mizo30	F_3_
8	AM-10 bulk	*A. esculentus* IC265650	*A. pungens* var. *mizoramensis* IC0624222	C_3_/50/mizo30 bulk	F_3_
9	AM-12	*A. esculentus* IC265650	*A. pungens* var. *mizoramensis* IC0624222	C_4_/50/mizo24	F_4_
10	AM-14	*A. esculentus* IC265650	*A. pungens* var. *mizoramensis* IC0624222	C_3_/50/mizo30	F_3_
11	AM-15	*A. esculentus* IC260106	*A. pungens* var. *mizoramensis* IC0624222	C_3_/106/mizo30	F_3_
12	AM-17 bulk	*A. esculentus* IC265650	*A. pungens* var. *mizoramensis* IC0624222	C_3_/50/mizo30 bulk	F_3_
13	AM-23	*A. esculentus* IC412987	*Abelmoschus angulosus* var. *grandiflorus* IC613527	C_3_/87/gran4	F_3_
14	AM-24	*A. esculentus* IC265650	*A. pungens* var. *mizoramensis* IC0624222	C_3_/50/mizo34	F_3_
15	AM-25	*A. caillei* IC566817	*A. angulosus* var. *grandiflorus* IC613527	C_3_/817/gran	F_3_
16	Ruchi × *Abelmoschus tetraphyllus*	*A. esculentus* cv. Ruchi	A. tetraphyllus	C_2_/Ruchitetra1	F_2_

Gn, generation.

**Table 2C:** Details of pedigree information of backcrossed amphidiploid derivatives.

S. no.	Cross	Female parent	Male parent	Gn
1	Ruchi× AM-24 (C_3_/50/mizo34)	*Abelmoschus esculentus* cv. Ruchi	AM-24 (*A. esculentus* IC265650 × *Abelmoschus pungens* var. *mizoramensis* IC0624222)	BC_1_F_3_
2	Ruchi × AM-6 (C_3_/50/mizo24)	*A. esculentus* cv. Ruchi	AM-6 (*A. esculentus* IC265650 × *A. pungens* var. *mizoramensis* IC0624222)	BC_1_F_3_
3	AM-24 (C_3_/50/mizo34) × Salkeerthi	*A. esculentus* IC265650 × *A. pungens* var. *mizoramensis* IC0624223	*A. esculentus* cv. Salkeerthi	BC_1_F_3_
4	C_3_/50/mizo27 × IC22232	*A. esculentus* IC265650 × *A. pungens* var. *mizoramensis* IC0624224	*A. esculentus* IC22232	BC_1_F_3_
5	AM-24 (C_3_/50/mizo34) × Parbhani Kranti	*A. esculentus* IC265650 × *A. pungens* var. *mizoramensis* IC0624225	*A. esculentus* cv. Parbhani Kranti	BC_1_F_3_
6	C_3_/741/mizo9 × EC169415	*A. esculentus* IC306741 × *A. pungens* var. *mizoramensis* IC0624226	*A. esculentus* EC169416	BC_1_F_3_
7	C_3_/50/mizo27 × Arka Anamika	*A. esculentus* IC265650 × *A. pungens* var. *mizoramensis* IC0624225	*A. esculentus* cv. Arka Anamika	BC_1_F_3_
8	C_3_/106/mizo6-(4) × IC22232	*A. esculentus* IC260106 × *A. pungens* var. *mizoramensis* IC0624225	*A. esculentus* IC22232	BC_1_F_3_
9	AM-6 (C_3_/50/mizo24) × IC31340C	*A. esculentus* IC265650 × *A. pungens* var. *mizoramensis* IC0624225	*A. esculentus* IC31340C	BC_1_F_3_
10	C_3_/50/mizo27 × IC31340C	*A. esculentus* IC265650 × *A. pungens* var. *mizoramensis* IC0624226	*A. esculentus* IC31340C	BC_1_F_3_
11	C_3_/87/gran2 × EC169415	*A. esculentus* IC412987 × *Abelmoschus angulosus* var. *grandiflorus* IC613527	*A. esculentus* EC169415	BC_1_F_3_
12	C_3_/741/gran12 × IC31398A	*A. esculentus* IC306741 × *A. angulosus* var. *grandiflorus* IC613527	*A. esculentus* IC31398A	BC_1_F_3_
13	C_3_/106/mizo6(4) × Kashi Vibhuti	*A. esculentus* IC260106 × *A. pungens* var. *mizoramensis* IC0624225	*A. esculentus* cv. Kashi Vibhuti	BC_1_F_3_
14	AM-24 (C_3_/50/mizo34) × South Canara Local	*A. esculentus* IC265650 × *A. pungens* var. *mizoramensis* IC0624225	*A. esculentus* cv. South Canara Local	BC_1_F_3_
15	C_3_/106/mizo6 (2) × IC22232	*A. esculentus* IC265650 × *A. pungens* var. *mizoramensis* IC0624225	*A. esculentus* IC22232	BC_1_F_3_
16	C_3_/50/mizo27 × IC31398A	*A. esculentus* IC265650 × *A. pungens* var. *mizoramensis* IC0624225	*A. esculentus* IC31398A	BC_1_F_3_
17	AM-24 (C_3/_50/mizo34) × Arka Anamika	*A. esculentus* IC265650 × *A. pungens* var. *mizoramensis* IC0624225	*A. esculentus* cv. Arka Anamika	BC_1_F_3_
18	C_3/_50/mizo27 × Kashi Vibhuti	*A. esculentus* IC265650 × *A. pungens* var. *mizoramensis* IC0624225	*A. esculentus* cv. Kashi Vibhuti	BC_1_F_3_
19	C_3_/741/gran12 × IC32398A	*A. esculentus* IC306741 × *A. angulosus* var. *grandiflorus* IC613527	*A. esculentus* IC32398A	BC_1_F_3_
20	C_3_/106/mizo6 (6) × Kashi Vibhuti	*A. esculentus* IC260106 × *A. pungens* var. *mizoramensis* IC0624225	*A. esculentus* cv. Kashi Vibhuti	BC_1_F_3_
21	C_3_/50/mizo24 × South Canara Local	*A. esculentus* IC265650 × *A. pungens* var. *mizoramensis* IC0624225	*A. esculentus* cv. South Canara Local	BC_1_F_3_
22	C_3_/50/mizo1 × IC31398A	*A. esculentus* IC265650 × *A. pungens* var. *mizoramensis* IC0624225	*A. esculentus* IC31398A	BC_1_F_3_
23	C_3_/50/mizo1 × Kashi Lalima	*A. esculentus* IC265650 × *A. pungens* var. *mizoramensis* IC0624225	*A. esculentus* cv. Kashi Lalima	BC_1_F_3_
24	C_3_/106/mizo6 × Hissar Unnat	*A. esculentus* IC260106 × *A. pungens* var. *mizoramensis* IC0624225	*A. esculentus* cv.Hissar Unnat	BC_1_F_3_
25	C_3_/87gran2 × Arka Anamika	*A. esculentus* IC412987 × *A. angulosus* var. *grandiflorus* IC613527	*A. esculentus* cv. Arka Anamika	BC_1_F_3_
26	B13 [(*A. esculentus* × *A. angulosus* var. *grandiflorus*) × (*A. esculentus* × *Abelmoschus tetraphyllus*)] × Arka Anamika	[*A. esculentus* IC265650 × *A. angulosus* var. *grandiflorus* IC613527] × [(*A. esculentus* cv. Arka Anamika × *A. tetraphyllus* IC253122)]	*A. esculentus* cv. Arka Anamika	BC_1_F_2_
27	B-16 [(*A. esculentus* × *A. angulosus* var. *grandiflorus*) × (*A. esculentus* × *A. tetraphyllus*)] × Arka Anamika	[*A. esculentus* IC265650 × *A. angulosus* var. *grandiflorus* IC613527] × [(*A. esculentus* Arka Anamika × *A. tetraphyllus* IC253122)]	*A. esculentus* Arka Anamika	BC_1_F_2_
28	Ruchi × AM-25 (C_2_/817/gran)	*A. esculentus* cv. Ruchi	*Abelmoschus caillei* IC566817 × *A. angulosus* var. *grandiflorus* IC613527	BC_1_F_2_
29	Arka Anamika × AMPK-1 [AM-24 (C_3_/50/mizo34) × Parbhani Kranti)]	*A. esculentus* Arka Anamika	*A. esculentus* IC265650 × *A. pungens* var. *mizoramensis* IC0624222 × *A. esculentus* cv. Parbhani Kranti	BC_2_F_3_
30	Ruchi × AMPK-1 [AM-24 (C_3_/50/mizo34) × Parbhani Kranti)]	*A. esculentus* cv. Ruchi	[*A. esculentus* IC265650 × *A. pungens* var. *mizoramensis* IC0624222] × *A. esculentus* cv. Parbhani Kranti	BC_2_F_3_

Gn, generation.

**Table 2D:** Details of pedigree information of crosses between amphidiploid derivatives.

S. no.	Cross	Female parent	Male parent
1	B-9 [(*Abelmoschus esculentus* × *Abelmoschus angulosus* var. *grandiflorus*) × (*A. esculentus* × *Abelmoschus tetraphyllus*)] × AM-24 (C_3_/50/mizo34)	[*A. esculentus* IC265650 × *A. angulosus* var. *grandiflorus* IC613527] × [(*A. esculentus* Arka Anamika × *A. tetraphyllus* IC253122)]	*A. esculentus* IC265650 × *Abelmoschus pungens* var. *mizoramensis* IC0624222
2	B-5 [(*A. esculentus* × *A. angulosus* var. *grandiflorus*) × (*A. esculentus* × *A. pungens* var. *mizoramensis*)] × AM-6 (C_3_/50/mizo24)	[*A. esculentus* IC265650 × *A. angulosus* var. *grandiflorus* IC613527] × [*A. esculentus* IC265650 × *A. pungens* var. *mizoramensis* IC0624222]	*A. esculentus* IC265650 × *A. pungens* var. *mizoramensis* IC0624222
3	B-2 [(WAS43 (*A. esculentus* × *A. tetraphyllus*) × Ruchi)] × AM-24 (C_3_/50/mizo34)	[(*A. esculentus* Arka Anamika × *A. tetraphyllus* IC253122)] × *A. esculentus* cv. Ruchi	*A. esculentus* IC265650 × *A. pungens* var. *mizoramensis* IC0624222
4	B-4 [(*A. esculentus* × *A. tetraphyllus*) × *Abelmoschus palianus*)] × AM-24 (C_3_/50/mizo34)	[(*A. esculentus* Arka Anamika × *A. tetraphyllus* IC253122)] × *A. palianus* IC0624218	*A. esculentus* IC265650 × *A. pungens* var. *mizoramensis* IC0624222
5	B-9 [(*A. esculentus* × *A. angulosus* var. *grandiflorus*) × (*A. esculentus* × *A. tetraphyllus*)] × AM-25 (C_3_/817/gran)	[*A. esculentus* IC265650 × *A. angulosus* var. *grandiflorus* IC613527] × [(*A. esculentus* Arka Anamika × *A. tetraphyllus* IC253122)]	AM25 (*Abelmoschus caillei* IC566817 × *A. angulosus* var. *grandiflorus* IC613527)
6	B-16 [(*A. esculentus* × *A. angulosus* var. *grandiflorus*) × (*A. esculentus* × *A. tetraphyllus*)] × AM-6 (C_3_/50/mizo24)	[*A. esculentus* IC265650 × *A. angulosus* var. *grandiflorus* IC613527] × [(*A. esculentus* Arka Anamika × *A. tetraphyllus* IC253122)]	*A. esculentus* IC265650 × *A. pungens* var. *mizoramensis* IC0624222
7	B-2 [(WAS43 (*A. esculentus* × *A. tetraphyllus*) × Ruchi)] × AM-6 (C_3_/50/mizo24)	[(*A. esculentus* Arka Anamika × *A. tetraphyllus* IC253122)] × *A. esculentus* cv. Ruchi	*A. esculentus* IC265650 × *A. pungens* var. *mizoramensis* IC0624222
8	B-16[(*A. esculentus* × *A. angulosus* var. *grandiflorus*) × (*A. esculentus* × *A. tetraphyllus*)] × AM-24 (C_3_/50/mizo34)	[*A. esculentus* IC265650 × *A. angulosus* var. *grandiflorus* IC613527] × [(*A. esculentus* Arka Anamika × *A. tetraphyllus* IC253122)]	*A. esculentus* IC265650 × *A. pungens* var. *mizoramensis* IC0624222
9	B-15 [(*A. esculentus* × *A. pungens* var. *mizoramensis*) × (*A. esculentus* × *A. tetraphyllus*)] × AM-25 (C_3_/817/gran)	[*A. esculentus* IC265650 × *A. pungens* var. *mizoramensis* IC0624222]× (*A. esculentus* Arka Anamika × *A. tetraphyllus* IC253122)]	AM25 (*A. caillei* IC566817 × *A. angulosus* var. *grandiflorus* IC613527)
10	B-13 [(*A. esculentus* × *A. angulosus* var. *grandiflorus*) × (*A. esculentus* × *A. tetraphyllus*)] × AM-6 (C_3_/50/mizo24)	[*A. esculentus* IC265650 × *A. angulosus* var. *grandiflorus* IC613527] × [(*A. esculentus* Arka Anamika × *A. tetraphyllus* IC253122)]	*A. esculentus* IC265650 × *A. pungens* var. *mizoramensis* IC0624222
11	B-15 [(*A. esculentus* × *A. pungens* var. *mizoramensis*) × (*A. esculentus* × *A. tetraphyllus*)] × AM-24 (C_3_/50/mizo34)	[*A. esculentus* IC265650 × *A. pungens* var. *mizoramensis* IC0624222] × [(*A. esculentus* Arka Anamika × *A. tetraphyllus* IC253122)]	*A. esculentus* IC265650 × *A. pungens* var. *mizoramensis* IC0624222
12	B-9 [(*A. esculentus* × *A. angulosus* var. *grandiflorus*) × (*A. esculentus* × *A. tetraphyllus*)] × AM-6 (C_3_/50/mizo24)	[*A. esculentus* IC265650 × *A. angulosus* var. *grandiflorus* IC613527] × [(*A. esculentus* Arka Anamika × *A. tetraphyllus* IC253122)]	*A. esculentus* IC265650 × *A. pungens* var. *mizoramensis* IC0624222
13	B-13 [(*A. esculentus* × *A. angulosus* var. *grandiflorus*) × (*A. esculentus* × *A. tetraphyllus*)] × AM-24 (C_3_/50/mizo34)	[*A. esculentus* IC265650 × *A. angulosus* var. *grandiflorus* IC613527] × [(*A. esculentus* Arka Anamika × *A. tetraphyllus* IC253122)]	*A. esculentus* IC265650 × *A. pungens* var. *mizoramensis* IC0624222
14	AM-24 (C_3_/50/mizo34) × B-4 [(*A. esculentus × A. tetraphyllus*) × *A. palianus*)]	*A. esculentus* IC265650 × *A. pungens* var. *mizoramensis* IC0624222]	(*A. esculentus* Arka Anamika × *A. tetraphyllus* IC253122) × *A. palianus* IC0624218]
15	AM-6 (C_3_/50/mizo24) × [(*A. esculentus* × *A. tetraphyllus*)]	*A. esculentus* IC265650 × *A. pungens* var. *mizoramensis* IC0624222	*A. esculentus* Arka Anamika × *A. tetraphyllus* IC253122
16	B-14 [(*A. esculentus* × *A. pungens* var. *mizoramensis*) × (*A. esculentus* × *A. tetraphyllus*)] × AM-6 (C_3_/50/mizo24)	[*A. esculentus* IC265650 × *A. pungens* var. *mizoramensis* IC0624222]× [(*A. esculentus* Arka Anamika × *A. tetraphyllus* IC253122)]	*A. esculentus* IC265650 × *A. pungens* var. *mizoramensis* IC0624222
17	B-13 [(*A. esculentus* × *A. angulosus* var. *grandiflorus*) × (*A. esculentus* × *A. tetraphyllus*)] × AM-25 (C_3_/817/gran)	[*A. esculentus* IC265650 × *A. angulosus* var. *grandiflorus* IC613527] × [(*A. esculentus* Arka Anamika × *A. tetraphyllus* IC253122)]	*A. caillei* IC566817 × *A. angulosus* var. *grandiflorus* IC613527
18	B-9 [(*A. esculentus* × *A. angulosus* var. *grandiflorus*) × (*A. esculentus* × *A. tetraphyllus*)] × AM-10 (C_3_/50/mizo30 bulk)	[*A. esculentus* IC265650 × *A. angulosus* var. *grandiflorus* IC613527] × [(*A. esculentus* Arka Anamika × *A. tetraphyllus* IC253122)]	*A. esculentus* IC265650 × *A. pungens* var. *mizoramensis* IC0624222
19	AM-23 (C_3_/87/gran4) × AM-6 (C_3_/50/mizo24)	[*A. esculentus* IC412987 × *A. angulosus* var. *grandiflorus* IC613527]	*A. esculentus* IC265650 × *A. pungens* var. *mizoramensis* IC0624222
20	B-12 [AM-13 (*A. esculentus* × *A. pungens* var. *mizoramensis*) × *A. palianus*] × AM-6 (C_3_/50/mizo24)	[*A. esculentus* IC265650 × *A. pungens* var. *mizoramensis* IC0624222] × *A. palianus*	*A. esculentus* IC265650 × *A. pungens* var. *mizoramensis* IC0624222
21	B-16 [(*A. esculentus* × *A. angulosus* var. *grandiflorus*) × (*A. esculentus* × *A. tetraphyllus*)] × AM-23 (C_3_/87/grand4)	[*A. esculentus* IC265650 × *A. angulosus* var. *grandiflorus* IC613527] × [(*A. esculentus* Arka Anamika × *A. tetraphyllus* IC253122)]	[*A. esculentus* IC265650 × *A. angulosus* var. *grandiflorus* IC613527]
22	B-2 [(WAS-43 (*A. esculentus* × *A. tetraphyllus*) × Ruchi)] × AM-23 (C_3_/87/gran4)	(*A. esculentus* Arka Anamika × *A. tetraphyllus* IC253122) × *A. esculentus* cv. Ruchi	[*A. esculentus* IC412987 × *A. angulosus* var. *grandiflorus* IC613527]
23	B-2 [(WAS-43 (*A. esculentus* × *A. tetraphyllus*) × Ruchi)] × AM-25 (C_3_/817/gran)	(*A. esculentus* Arka Anamika × *A. tetraphyllus* IC253122) × *A. esculentus* cv. Ruchi	*A. caillei* IC566817 × *A. angulosus* var. *grandiflorus* IC613527
24	B-14 [(*A. esculentus* × *A. pungens* var. *mizoramensis*) × (*A. esculentus* × *A. tetraphyllus*)] × AM-25 (C_3_/817/gran)	[*A. esculentus* IC265650 × *A. pungens* var. *mizoramensis* IC0624222] × [(*A. esculentus* Arka Anamika × *A. tetraphyllus* IC253122)]	*A. caillei* IC566817× *A. angulosus* var. *grandiflorus* IC613527
25	B-9 [(*A. esculentus* × *A. angulosus* var. *grandiflorus*) × (*A. esculentus* × *A. tetraphyllus*)] × AM-23 (C_3_/87/gran4)	*[A. esculentus* IC265650 × *A. angulosus* var. *grandiflorus* IC613527] × [(*A. esculentus* Arka Anamika × *A. tetraphyllus* IC253122)]	[*A. esculentus* IC412987 × *A. angulosus* var. *grandiflorus* IC613527]
26	AM-25 (C_3_/817/gran) × CR-1 [(*A. esculentus* × *A. sagittifolius*)]	*A. caillei* IC566817 × *A. angulosus* var. *grandiflorus* IC613527	CR-1 [*A. esculentus* IC260106 × *A. sagittifolius* IC470750]
27	B-4 [(*A. esculentus* × *A. tetraphyllus*) × *A. palianus*)] × WAS-15 (*A. enbeepeegearensis*)	(*A. esculentus* Arka Anamika × *A. tetraphyllus* IC253122) × *A. palianus* IC0624218	WAS-15 (*A. enbeepeegearensis* IC582757)

**Table 2E:** Details of pedigree information of multi-cross combinations.

S. no.	Cross	Female parent	Male parent
1	B-16 [(*Abelmoschus esculentus* × *Abelmoschus angulosus* var. *grandiflorus*) × (*A. esculentus* × *Abelmoschus tetraphyllus*)]	[*A. esculentus* IC265650 × *A. angulosus* var. *grandiflorus* IC613527]	(*A. esculentus* Arka Anamika × *A. tetraphyllus* IC253122)
2	B-15 [(*A. esculentus* × *Abelmoschus pungens* var. *mizoramensis*) × (*A. esculentus* × *A. tetraphyllus*)]	*A. esculentus* IC265650 × *A. pungens* var. *mizoramensis* IC0624222	(*A. esculentus* Arka Anamika × *A. tetraphyllus* IC253122)
3	B-13 [(*A. esculentus* × *A. angulosus* var. *grandiflorus*) × (*A. esculentus* × *A. tetraphyllus*)]	[*A. esculentus* IC265650 × *A. angulosus* var. *grandiflorus* IC613527]	(*A. esculentus* Arka Anamika × *A. tetraphyllus* IC253122)
4	B-9 [(*A. esculentus* × *A. angulosus* var. *grandiflorus*) × (*A. esculentus* × *A. tetraphyllus*)]	[*A. esculentus* IC265650 × *A. angulosus* var. *grandiflorus* IC613527]	(*A. esculentus* Arka Anamika × *A. tetraphyllus* IC253122)
5	B-6 [(*A. esculentus* × *A. angulosus* var. *grandiflorus*) × (*A. esculentus* × *A. pungens* var. *mizoramensis*)]	[*A. esculentus* IC265650 × *A. angulosus* var. *grandiflorus* IC613527]	[*A. esculentus* IC265650 × *A. pungens* var. *mizoramensis* IC0624222
6	B-5 [(*A. esculentus* × *A. angulosus* var. *grandiflorus*) × (*A. esculentus* × *A. pungens* var. *mizoramensis*)]	[*A. esculentus* IC265650 × *A. angulosus* var. *grandiflorus* IC613527]	[*A. esculentus* IC265650 × *A. pungens* var. *mizoramensis* IC0624222
7	B-4 [(*A. esculentus* × *A. tetraphyllus*) × *A. palianus*)]	(*A. esculentus* Arka Anamika × *A. tetraphyllus* IC253122)	*A. palianus* IC0624218

**Table 2F:** Details of pedigree information of interspecific cross derivatives at F8 generation.

S. no.	Amphidiploid code	Female parent	Male parent	Gn
1	AM-7	*Abelmoschus sagittifolius* IC470750	*Abelmoschus moschatus* subsp. *moschatus* IC0624232	F_8_
2	AM-13	*A. sagittifolius* IC470750	*A. moschatus* subsp. *moschatus* IC0624232	F_8_
3	AM-19	*A. sagittifolius* IC470750	*A. moschatus* subsp. *moschatus* IC0624232	F_8_

Gn, generation.

Hence, in the present study, the amphidiploids developed over the years in various generations included a total of 113 derivatives comprising amphidiploids in the F_1_ generation (30); F_3_ (14); one each in the F_2_ and F_4_ generations; backcross generation in BC_1_F_2_ (03), BC_1_F_3_ (25), and BC_2_F_3_ (02); crosses between amphidiploids (27); multi-cross combinations (07); and interspecific cross [between *A. sagittifolius* (Kurz) Merr. (Syn. *A. moschatus* subsp. *tuberosus* Borss.Waalk.) and *A. moschatus* subsp. *moschatus*] selfed derivatives at the F_8_ generation (03). Polyploidization using colchicine treatment was not undertaken in the cross between *A. sagittifolius* and *A. moschatus* subsp. *moschatus*, as seed setting (few) was observed in this cross. The pedigree information of the amphidiploids is given in **Tables 2A–F**. Data were taken for 11 quantitative (flower and fruit characteristics) and one qualitative [(presence of spiny hairs scored as absent (0), soft hairs (1), and spiny hairs (2)] characteristics following Charrier (1984) and minimal descriptors for okra (Srivastava et al., 2001). Observations on general morphology (growth habit, branching pattern, nature of lobing of leaves, and presence of red/purple color on the plant parts) were also taken. The diverse amphidiploids were raised in the field from June to November 2021 at ICAR-NBPGR, Regional Station, Vellanikkara, Thrissur, Kerala, India, located at 10.5480°N, 76.2830°E, to understand the variability pattern, to identify desirable segregants, and for generation advancement. The temperature during the crop period was between 23.4°C and 32.5°C, with a mean rainfall ranging from 19.20 mm to 626.9 mm and with an average of 18.16 rainy days per month. The data were analyzed using Microsoft Excel 2019, and summary statistics for each category of amphidiploids (mean, range, standard deviation, and coefficient of variation) were derived.

## Results

**Figure 4:**
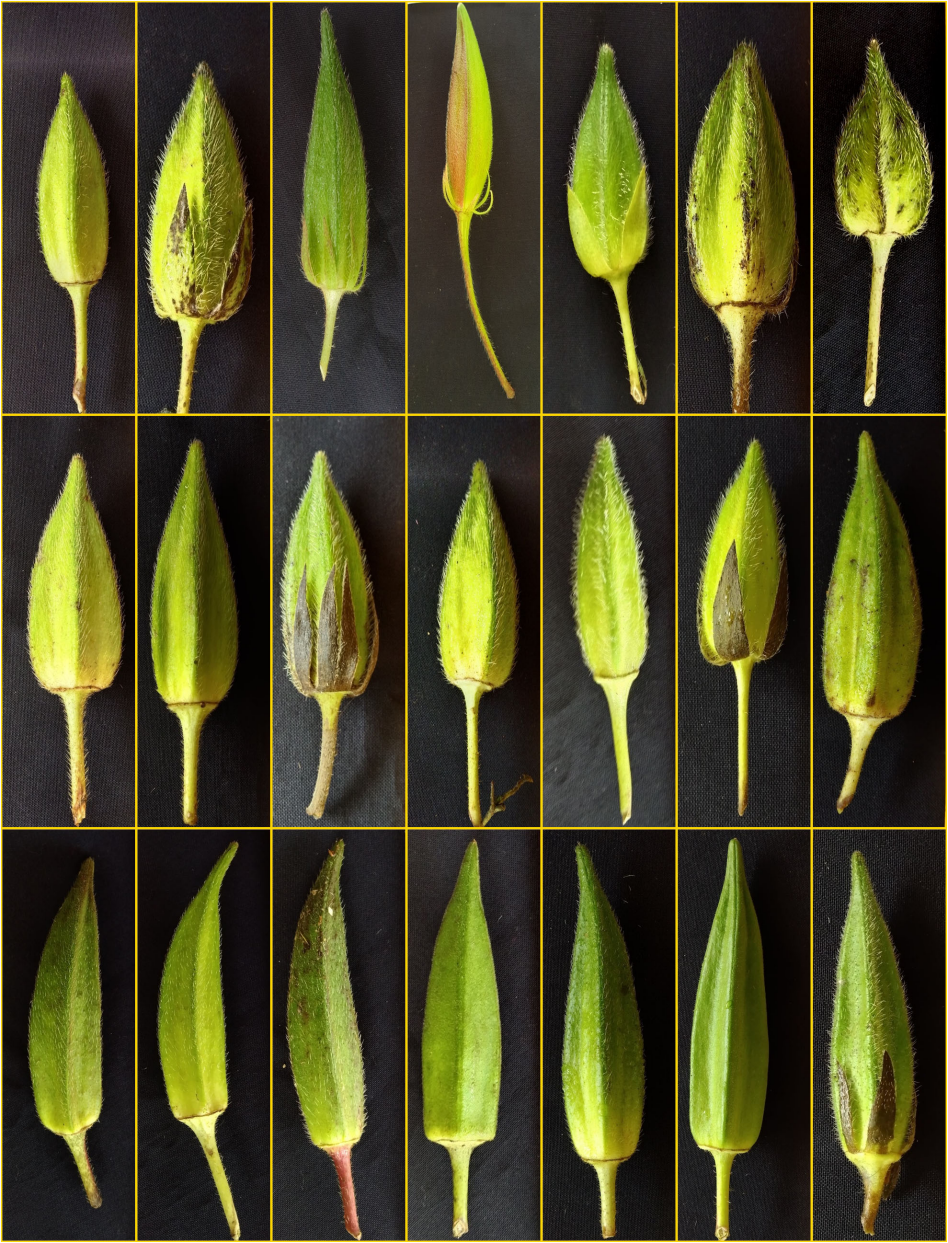
Variability in fruits of various amphidiploid derivatives.

Interspecific hybridization followed by polyploidization resulted in diverse amphidiploids, which in general exhibited intermediate phenotypic traits with dominant wild characteristics (**Figure 4**). Wild *Abelmoschus* species differ from the cultivated types, with respect to the growth habit, branching pattern, density of indumentum (hairiness over plant parts), length and width of the epicalyx, persistence of epicalyx at maturity, fruit length, and growth duration (perennial nature). For reference, **Table 3** shows the characteristics of various *Abelmoschus* spp. used in this study. Basal orthotropic branching; shallow lobing of the older leaves (at bottom) and deep lobing of the top leaves; reddish coloration on the stems, petioles, and nodal region; and yellow flowers were the frequently observed characteristics in the amphidiploids. Additionally, prolific and extended bearing and densely hirsute fruits were regularly observed. The number of ridges on the fruit surface was invariably five in all the derivatives.

**Table 3:** Characteristics of wild *Abelmoschus* species used in wide hybridization program.

Species	Growth habit	FL (cm)	FW (cm)	NEpS	NR	FPL (cm)	FS	EpCL (cm)	EpCW (cm)	SEpCS	FwD (cm)	Hairiness on fruit
*Abelmoschus esculentus*	Annual herb	7.0–30.0	2.0–3.0	6.0–10.0	5	2.5–3.5	Long fusiform	2.1–2.4	0.5–0.6	Linear	6.0–7.0	Tomentulose
*Abelmoschus caillei*	Annual or biannual herb	6.0–18.0	2.0–4.0	7.0–9.0	5	2.3–2.6	Lanceolate to lance-ovate	1.0–3.5	0.4–1.5	Ovate-deltoid	6.0–10.0	Tomentulose
*A. pungens* var. *mizoramensis*	Perennial shrub	3.5–5.0	2.5–3.0	4.0–5.0	5	2.4–2.6	Ovoid	2.3–2.5	1.0–1.3	Lanceolate	7.0–8.0	Densely hirsute
*Abelmoschus tetraphyllus*	Annual subshrub	3.5–4.5	1.4–1.5	4.0–5.0	5	2.0–2.9	Oblong to ovoid	2.3–2.2.7	0.9–1.3	Ovate-cordate	5.0–7.0	Pubescent
*Abelmoschus angulosus* var. *grandiflorus*	Annual subshrub	3.0–4.0	1.3–1.5	4.0	5	2.3–2.5	Ovoid to oblong	2.0–2.5	1.3–1.5	Deltoid	5.0–7.0	Densely hispid
*Abelmoschus enbeepeegearensis*	Perennial herb	3.5–4.1	2.5–3.0	10.0–11.0	5	4.3–4.5	Ovate	2.0–3.5	0.1–0.2	Linear	6.0–9.0	Soft strigulose
*Abelmoschus palianus*	Annual herb	3.5–4.0	2.5–2.7	5.0–7.0	5	2.0–4.0	Broadly ovate	1.8–2.0	0.3–0.5	Ovate to narrowly ovate	7.0–8.0	Densely hirsute
*Abelmoschus moschatus* subsp. *moschatus*	Annual undershrub	5.0–6.0	2.0–2.5	7.0–8.0	5	6.0–15.3	Ovoid or globose	0.8–0.9	0.1	Linear	7.0–9.0	Soft strigulose
*Abelmoschus sagittifolius*	Perennial herb	4.5–5.0	2.5–3.2	10.0–11.0	5	6.5–13.3	Ovoid or globose	2.0–2.6	0.1–0.2	Linear	8.0–10.0	Soft strigulose

FL, fruit length; FW, fruit width; NEpS, no. of epicalyx segments; NR, no. of ridges; FPL, fruit peduncle length; FS, fruit shape; EpCL, epicalyx length; EpCW, epicalyx width; SEpCS, shape of epicalyx segment; FwD, flower diameter.

Source: John et al., 2013c; Sutar et al., 2013; Yadav et al., 2014; Pandey et al., 2016.

### Selfed amphidiploid derivatives

**Figure 5:**
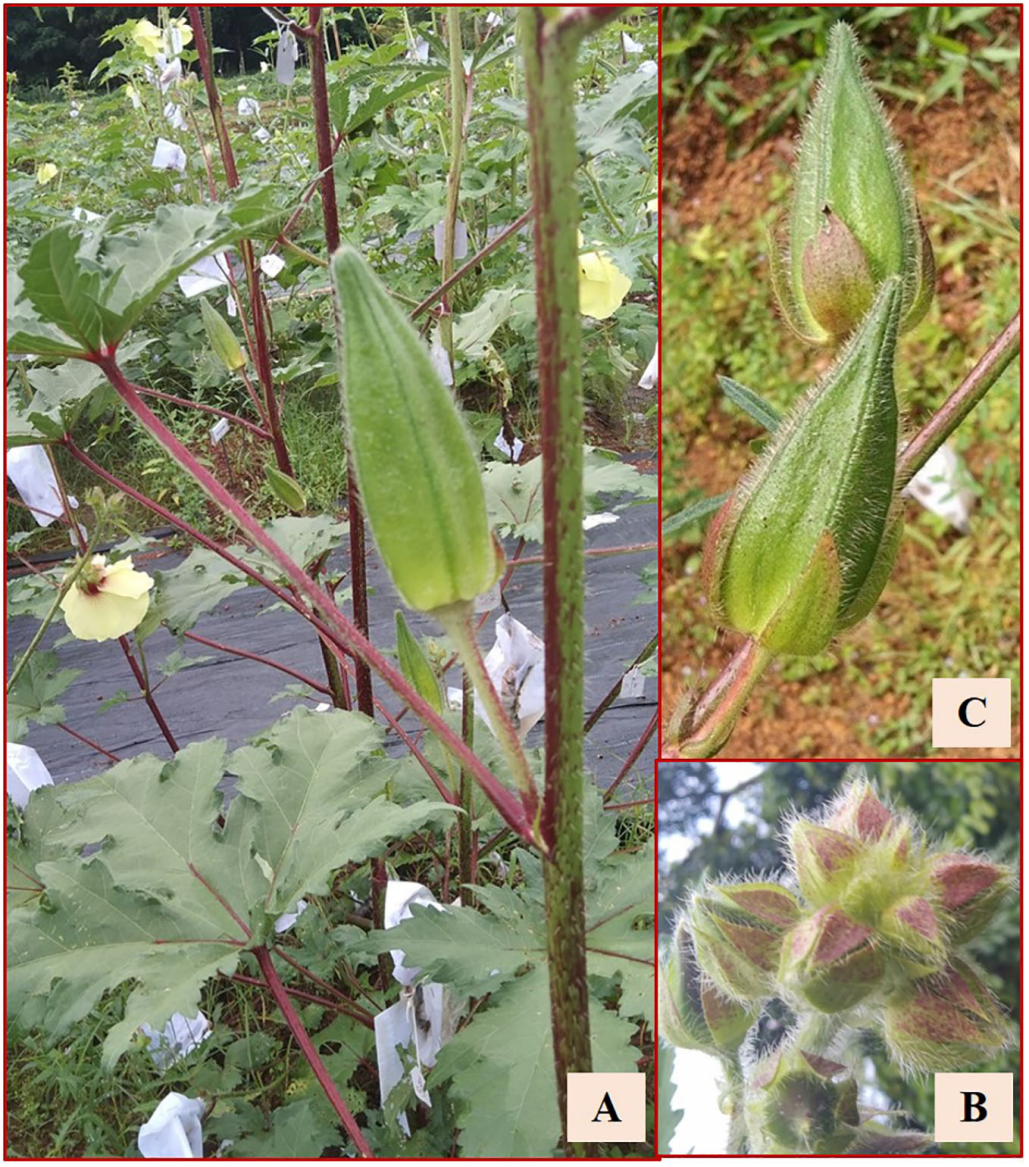
**(A)** Reddish coloration on stems and petioles. **(B)** Clustered flower buds in C_1_Pusamizo derivatives. **(C)** Hispid fruits.

Thirty out of the 113 crosses were amphidiploids in the F_1_ generation. Twenty-six were crossed between Pusa Sawani and *A. pungens* var. *mizoramensis* (IC624236 and IC624235 in 20 and 6 crosses, respectively) and four between Pusa Sawani and *A. moschatus* variant (EC306750). In all the above crosses, Pusa Sawani was the female parent. Of them, the plants of the amphidiploid derivatives with *A. pungens* var. *mizoramensis* as male parent exhibited similar morphology. The stems, petioles, and fruits were highly hispid, and a reddish color was observed on the stems and petioles. Further, the flower buds of C_1_Pusamizo236 and C_1_Pusamizo235 derivatives were clustered at the top, resembling the wild parent *A. pungens* var. *mizoramensis* (**Figures 5A–C**). The fruit length and width ranged from 6.13 cm (C_1_Pusamizo236-26) to 8.83 cm (C_1_Arkamosc (variant)-750-04) and 2.03 cm (C_1_Pusamizo236-02) to 2.93 cm (C_1_Pusamizo236-18), respectively (**Table 4A**). The epicalyces were medium broad (0.69 cm) and became withered/detached from the fruit at maturity. The majority of them possessed spiny hairs on the fruits. However, the fruits of C_1_Pusamizo236-14, C_1_Pusamizo236-02, C_1_Pusamizo236-30, C_1_Pusamizo235-09, C_1_Pusamizo235-02, and C_1_Pusamizo23-06 were characterized by soft spiny hairs (**Supplementary Table 1A**). As there was a wide range in the number of seeds per fruit among the C_1_Pusamizo derivatives (8.50 to 98.25), the coefficient of variation (CV) was high (49.90%) compared to other characteristics.

**Table 4A:** Summary statistics of the selfed derivatives comprising F_1_, F_2_, F_3_, and F_4_ generations.

	F_1_ generation				F_2_, F_3_, and F_4_ generations			
	Range	Mean	SD	CV (%)	Range	Mean	SD	CV (%)
FL (cm)	6.13–8.83	7.31	0.71	9.78	6.04–8.96	7.6	0.91	11.92
FW (cm)	2.03–2.93	2.39	0.24	9.96	1.5–2.80	2.45	0.31	12.49
NEpS	4.33–9.00	6.46	0.97	15.01	4.00–7.67	6.46	0.94	14.58
FPL (cm)	2.1–4.48	2.73	0.56	20.42	2.32–4.14	3.29	0.47	14.23
NSPF	8.5–98.25	31.94	15.94	49.9	9.00–52.75	26.48	13.37	50.49
EpCL (cm)	1.5–3.17	2.13	0.43	20.25	2.07–2.93	2.56	0.25	9.74
EpCW (cm)	0.17–0.97	0.69	0.19	27.4	0.5 0–1.60	0.81	0.24	29.55
PL (cm)	6.6–9.57	7.78	0.74	9.48	7.27–10.00	8.63	0.72	8.34
PW (cm)	6.23–9.77	8.15	0.86	10.52	7.33–9.80	8.74	0.8	9.11
FwD (cm)	10.47	12.08	0.84	6.97	10.30–13.93	12.34	0.97	7.88

FL, fruit length; FW, fruit width; NEpS, no. of epicalyx segments; NR, no. of ridges; FPL, fruit peduncle length; NSPF, no. of seeds per fruit; EpCL, epicalyx length; EpCW, epicalyx width; PL, petal length; PW, petal width; FwD, flower diameter.

**Table 4B:** Summary statistics of the BC derivatives comprising BC1F3, BC1F2, and BC2F3 generations.

Characteristics	BC_1_F_3_ generation				BC_1_F_2_ and BC_2_F_3_ generations			
	Range	Mean	SD	CV (%)	Range	Mean	SD	CV (%)
FL (cm)	3.80–13.60	9.23	2.22	24.03	6.02–11.40	7.49	2.26	30.16
FW (cm)	1.60–2.90	2.30	0.35	15.05	2.00–2.68	2.34	0.28	11.90
NEpS	5.00–10.00	7.21	0.97	13.42	5.67–8.00	6.60	0.92	14.01
FPL (cm)	1.55–4.50	3.44	0.69	20.17	2.60–3.76	3.02	0.52	17.06
NSPF	4.00–46.75	15.86	11.58	72.97	4.33–60.00	24.03	22.67	94.32
EpCL (cm)	1.40–3.93	2.62	0.43	16.52	1.80–2.53	2.18	0.32	14.77
EpCW (cm)	0.10–0.80	0.57	0.17	29.56	0.73–0.97	0.84	0.09	11.01
PL (cm)	4.30–10.97	8.35	1.47	17.61	6.80–9.67	8.20	1.31	16.03
PW (cm)	3.60–9.93	8.36	1.53	18.25	6.50–10.00	7.87	1.46	18.56
FwD (cm)	5.90–15.17	11.96	2.25	18.79	9.30–13.40	11.43	1.98	17.33

FL, fruit length; FW, fruit width; NEpS, no. of epicalyx segments; NR, no. of ridges; FPL, fruit peduncle length; NSPF, no. of seeds per fruit; EpCL, epicalyx length; EpCW, epicalyx width; PL, petal length; PW, petal width; FwD, flower diameter.

**Table 4C:** Summary statistics of the crosses between amphidiploid derivatives, multi-cross combinations, and interspecific cross selfed derivatives.

Characteristics	Crosses between amphidiploid derivatives				Multi-cross combinations				Interspecific cross selfed derivatives			
	Range	Mean	SD	CV (%)	Range	Mean	SD	CV (%)	Range	Mean	SD	CV (%)
FL (cm)	3.30–9.29	6.41	1.43	22.40	3.80–9.70	7.61	2.62	34.5	7.82–8.56	7.95	0.55	6.94
FW (cm)	1.73–3.15	2.40	0.35	14.74	2.10–2.55	2.34	0.17	7.254	2.53–2.63	2.50	0.14	5.57
NEpS	5.00–10.00	6.41	1.09	16.97	5.00–9.00	6.76	1.27	18.82	7.33–9.67	8.83	1.30	14.74
FPL (cm)	1.40–4.90	3.28	0.76	23.04	2.80–4.08	3.41	0.54	16.02	4.30–5.22	4.79	0.46	9.66
NSPF	7.33–33.25	18.49	8.56	46.30	8.50–24.33	16.52	6.52	39.48	11.00–85.25	56.17	39.65	70.60
EpCL (cm)	1.20–2.97	2.41	0.43	18.00	2.40–3.07	2.71	0.23	8.40	1.73–2.40	2.18	0.38	17.67
EpCW (cm)	0.13–1.17	0.80	0.24	30.66	0.43–1.10	0.71	0.24	34.17	0.17–0.73	0.38	0.31	79.82
PL (cm)	4.90–10.10	8.45	1.35	15.99	6.90–9.37	8.49	0.82	9.746	7.43–8.80	8.06	0.69	8.56
PW (cm)	3.77–10.53	8.52	1.73	20.27	7.20–10.37	8.71	0.98	11.32	5.70–8.40	7.00	1.35	19.33
FwD (cm)	5.70–16.70	12.04	2.73	22.65	9.20–14.80	11.76	1.68	14.3	10.77–13.05	11.74	1.18	10.04

FL, fruit length; FW, fruit width; NEpS, no. of epicalyx segments; NR, no. of ridges; FPL, fruit peduncle length; NSPF, no. of seeds per fruit; EpCL, epicalyx length; EpCW, epicalyx width; PL, petal length; PW, petal width; FwD, flower diameter.

**Figure 6:**
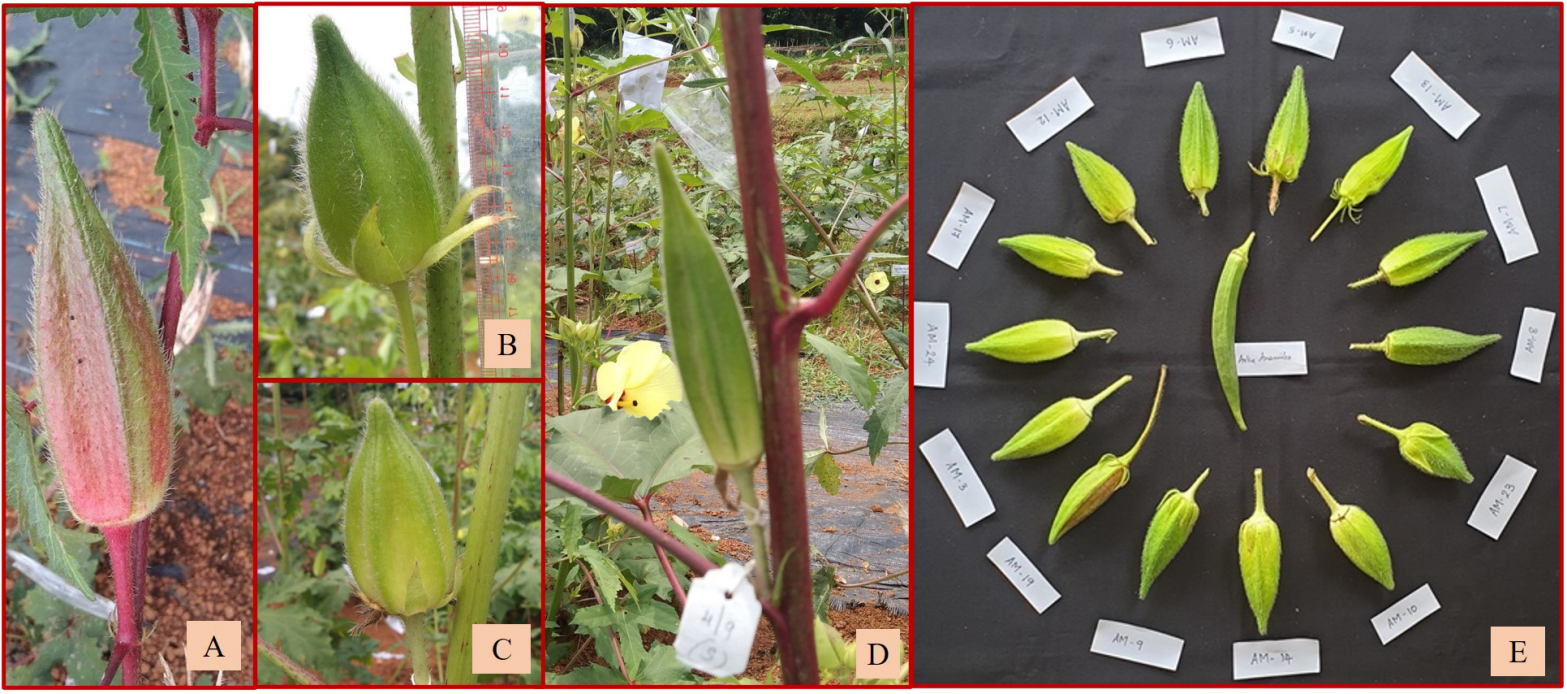
Fruit variability in selfed derivatives of F_2_, F_3_, and F_4_ generation. **(A)** Reddish fruits of AM-8. **(B)** Short fruit of AM-23. **(C)** Soft and spongy fruit of AM-3. **(D)** Fruit of C_2_/Ruchi/tetra1 with intermediate fruit length. **(E)** General variability in fruits of selfed derivatives.

Out of the remaining 16 amphidiploids, 14 were in the F_3_, and there was one each in the F_2_ and F_4_ generations. Among them, seven were progenies selected from the amphidiploid C_3_/50/mizo30 and three from C_3_/106/mizo30. Further, one derivative each from C_3_/50/mizo24, C_4_/50/mizo24, C_3_/50/mizo34, C_3_/87/gran4, C_3_/817/gran, and C_2_Ruchitetra (**Table 2B**) were also included. The *A. esculentus* genotype used in 10 amphidiploids was IC265650 (C_3_/50/mizo30 derivatives) and IC260106 in three (C_3_/106/mizo30) derivatives. IC412987 was the *A. esculentus* genotype in C_3_/87/gran4 and Ruchi in C_2_Ruchitetra1. An erect tall growth habit with basal branching, reddish streaks on the stems, and profuse growth were common characteristics. They also exhibited extended life span, prolific flowering and fruit set, and medium-long spiny ovoid elongate fruits. The fruits of AM-8 were reddish with spiny hairs (**Figure 6A**), which possessed the maximum length (8.96 cm) among the selfed derivatives. The fruit width was maximum in AM-23 (C_3_/87/gran4), measuring a mean value of 2.80 cm (**Figure 6B**) (**Supplementary Table 1B**). However, the young fruit surface was soft with a spongy nature in AM-3, a segregant of C_3_/106/mizo30 (**Figure 6C**). C_2_/Ruchi/tetra1 exhibited an intermediate fruit length (7.25 cm) with a fusiform shape (**Figure 6D**). AM-25 (C_3_/817/gran) was the only derivative with *A. caillei* as the female parent. The plants of this derivative exhibited green stems, red color at the nodes, broad epicalyces (0.77 cm), and fruits with a mean length of 6.23 cm. The number of seeds per fruit ranged from 9 (AM-23) to 53 (AM-3). Seed shattering was also observed in these amphidiploids. General variability in fruit characteristics among selected selfed derivatives is depicted in **Figure 6E**.

### Backcrossed derivatives

**Figure 7:**
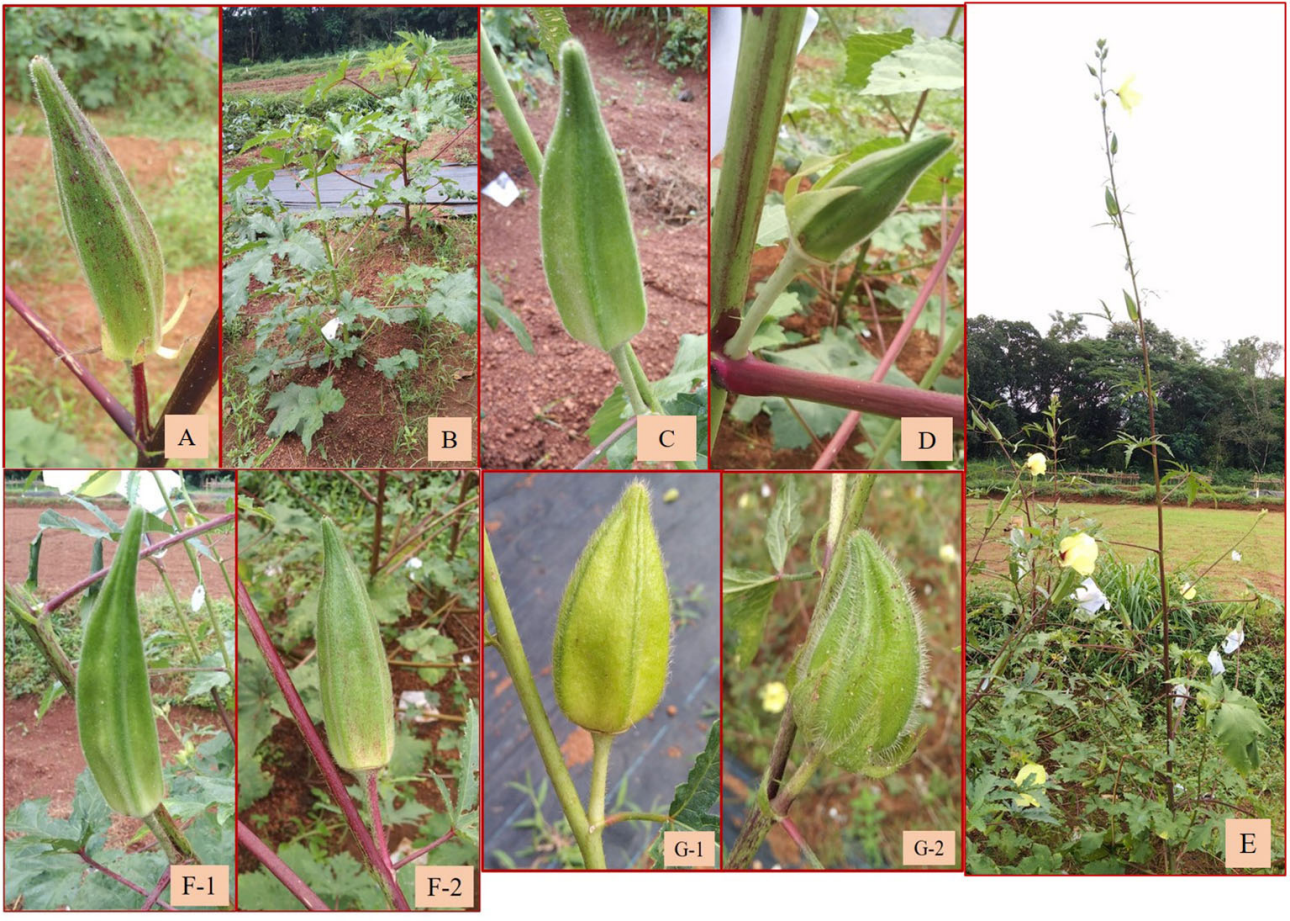
**(A)** Fruit of C_3_/50/mizo34 × Parbhani Kranti. **(B)** Segregation in stem color in C_3_/50/mizo34 **×** Salkeerthi. **(C)** Medium-long fruits of C_3_/50/mizo1 **×** IC31398A. **(D)** Velvety fruits of C_3_/50/mizo34 **×** IC22232. **(E)** Tall growth habit of C_3_/106/mizo6 **×** Hissar Unnat. **(F-1, F-2)** Fruits of C_3_/106/mizo6 **×** Kashi Vibhuti and C_3_/106/mizo6 **×** Hissar Unnat with low trichome density. **(G-1, G-2)** Short fruits of B16 **×** Arka Anamika and B13 **×** Arka Anamika.

Twenty-five backcrossed derivatives that developed (BC_1_F_3_) also exhibited variability with respect to the growth habit, branching behavior, density of indumentum, and leaf and fruit characteristics. The number of seeds per fruit was the highly variable characteristic indicated by the high CV of 72.97% (**Table 4B**; **Supplementary Table 1C**). Fruit length ranged from 3.80 cm (short fruit length resembling wild types) to 13.60 cm (resembling cultivated types) with a mean of 9.23 cm (**Table 4B**). The plants of C_3_/50/mizo34 **×** Parbhani Kranti had purplish stems, petioles with red dorsal and green ventral sides, intermediate epicalyces (0.50 cm), and medium-long fruits (10.75 cm) (**Figure 7A**). Additionally, they also possessed mildly lobed leaves at the bottom and deeply lobed at the top, and hispid stems and leaves. However, in C_3_/50/mizo34 **×** Salkeerthi, the plants exhibited segregation with respect to stem color (purplish green and purple stems) (**Figure 7B**). They also exhibited hispid stems, petioles, and fruits; intermediate epicalyx width (0.57 cm); and prominent basal branching. Nonetheless, tall plants with green stems having light reddish patches and the absence of basal branching were the characteristic features of C_3_/50/mizo1 **×** IC31398A. It produced medium-long fruits (7.70 cm) (**Figure 7C**) with soft hairs. Reddish purple patches on the stems and petioles, hispid stems, petioles and leaves, and red epicalyces were observed in C_3_/50/mizo34 **×** South Canara Local and C_3_/50/mizo34 **×** South Canara Local. A similar kind of reddish coloration on the stems, petioles, and epicalyces was observed in plants of C_3_/50/mizo34 **×** Arka Anamika. C_3_/741/mizo9 **×** EC169415, C_3_/50/mizo27 **×** Arka Anamika, and C_3_/106 mizo6(4) **×** IC22232 also exhibited wild characteristics like basal branching and late flowering. In contrast to the above, the fruits of C_3_/50/mizo27 **×** IC22232 were velvety (**Figure 7D**), with wide epicalyces (0.80 cm), even though IC22232 and IC265650 (accessions involved in the cross) belong to the *A. esculentus* (with linear epicalyx) taxon.

**Figure 8:**
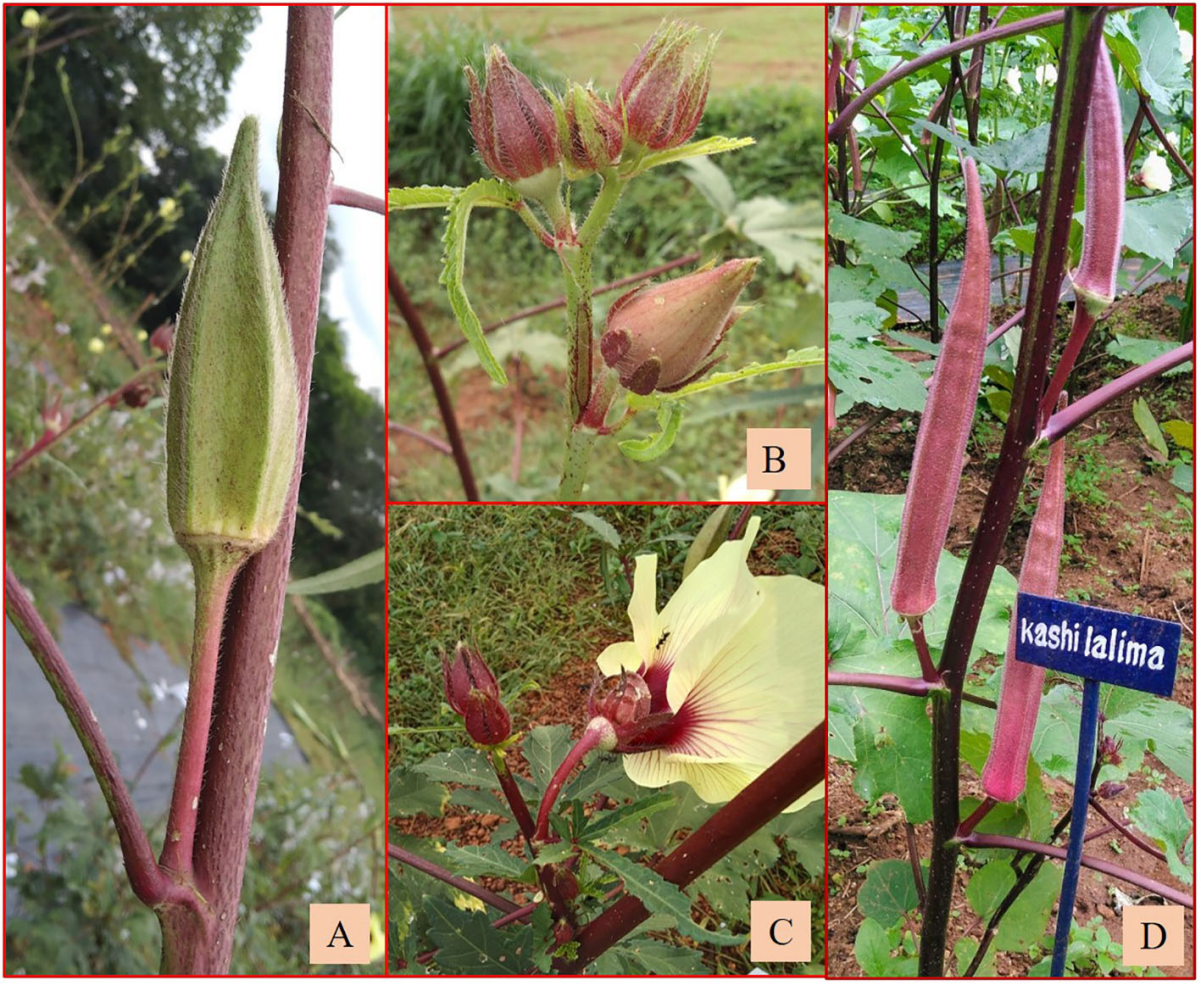
**(A)** Fruit. **(B)** Flower bud with epicalyx. **(C)** Flower base of C_3_/50/mizo1 **×** *A.esculentus* cv. Kashi Lalima. **(D)** Kashi Lalima.

Similarly, a robust tall growth habit, green stem, reddish spots and streaks along the stems, and reddish color at the nodes were observed among the plants of the derivatives C_3_/87/gran2 **×** EC169415. Epicalyx was broader (0.60 cm) and non-persistent; fruits were longer than those of the other amphidiploids (12.33 cm). It also exhibited mildly lobed leaves at the base and deeply lobed toward the top. C_3_/741/gran12 **×** IC31398A also had stems, petioles, fruits, and leaves covered with thick spiny hairs. Leaf lobing was like the former one, with red coloration at the node, with an average of 19 seeds per fruit; however, epicalyx persisted up to a certain maturity of the fruit. Branching extended up to half the height of the plants. The plants of C_3_/106/mizo6 × Hissar Unnat were also very tall with basal branching (**Figure 7E**), reddish stems and petioles, and prominent spiny hairs on them. The tall nature, robust growth, and low trichome density in fruits (**Figures 7F-1, F-2**) were common in all derivatives involving C_3_/106/mizo6. The fruit hairs were soft in B-16 **×** Arka Anamika and B-13 **×** Arka Anamika (BC_1_F_2_) (**Figures 7G-1, G-2**). C_3_/50/mizo1 **×** Kashi Lalima exhibited reddish coloration on the stems, epicalyces, flower buds, and petioles but with green hispid fruits and medium broad epicalyces (**Figures 8A–C**). The plants of derivative C_3_/87/gran2 **×** Arka Anamika were an intermediate type with a semi-dwarf habit but with highly spiny and reddish stems, basal branching, medium-long fruits (8.53 cm), and medium broad epicalyces (0.43 cm). However, the fruit surface was soft and hairy. Non-spiny fruit surface was observed in four amphidiploids (Ruchi **×** AM-24, Ruchi **×** AM-6, C_3_/50/mizo27 **×** IC22232, and C_3_/50/mizo27 **×** IC31398A). The fruits of the first two resembled the morphology of cultivated okra having mean fruit length of 12.55 and 13.60 cm and with slender linear epicalyces. The number of seeds per fruit was 40 in Ruchi **×** AM-24 and 24 in Ruchi **×** AM-6.

**Figure 9:**
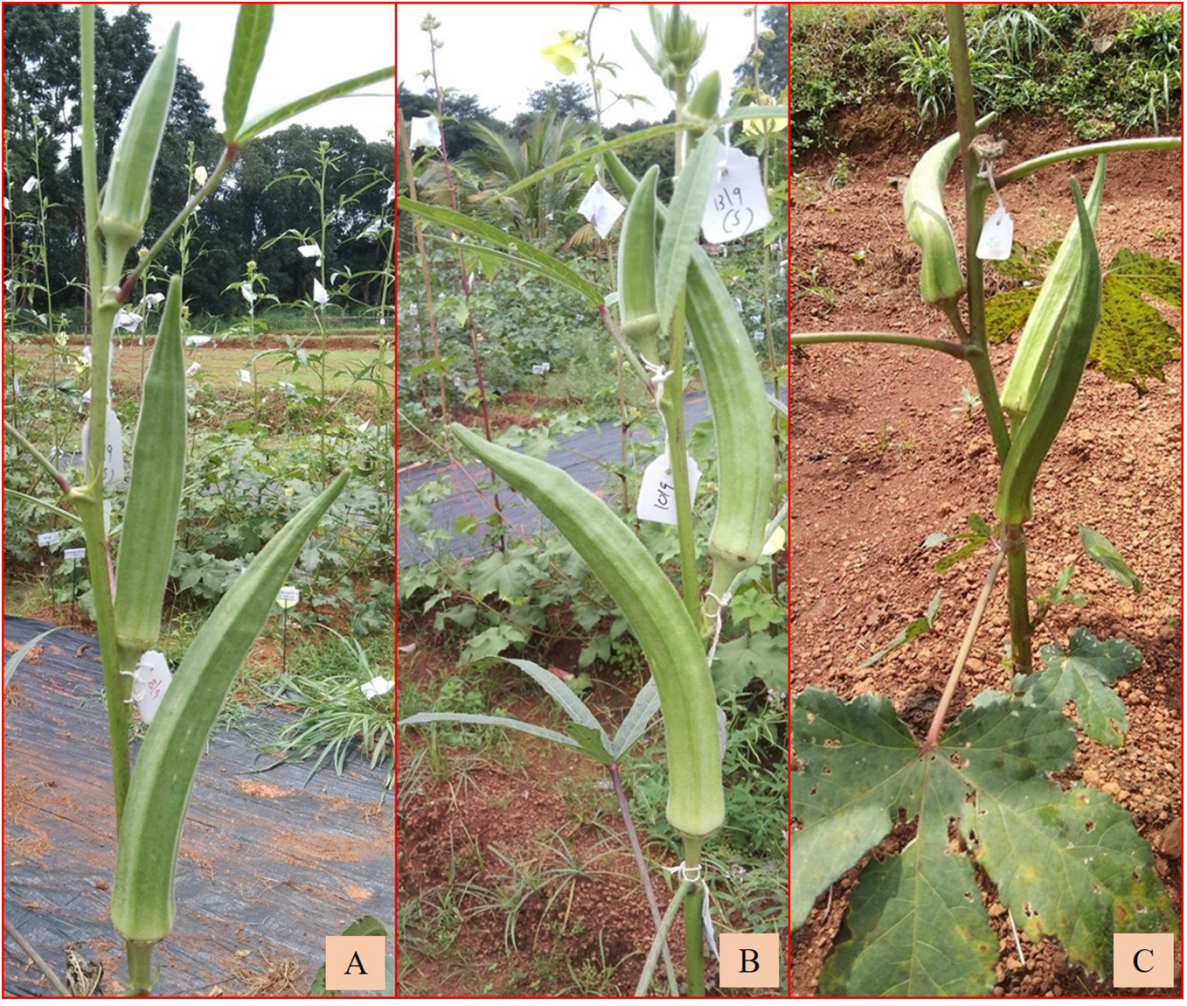
Backcrossed derivatives with cultivated okra morphology **(A)** Ruchi **×** AM-6. **(B)** Ruchi **×** AM-24. **(C)** Arka Anamika **×** AMPK-1.

Among the derivatives in the BC_1_F_2_ and BC_2_F_3_ generations, Ruchi **×** AMPK-1, Ruchi **×** AM25, B-13 **×** Arka Anamika, and B-16 **×** Arka Anamika were wild types with short to medium fruits (6.02 cm–7.46 cm) (**Supplementary Table 1D**) with the presence of soft to nil hairs. However, in B-16 **×** Arka Anamika, fewer branching was a distinct characteristic. Arka Anamika **×** AMPK-1 (BC_2_F_3_) exhibited morphology of cultivated *A. esculentus* with long fruits (11.40 cm) devoid of spiny hairs on the fruit, with the number of seeds as high as 60 per fruit. Narrow variability was exhibited among the derivatives for epicalyx width (0.73 cm–0.97 cm) and fruit width (2.00–2.68 cm) as demonstrated by the narrow range and low CV of 11.01% and 11.90%, respectively (**Table 4B**). However, the range of seed setting in fruits was wide (4.33–60.00). **Figures 9A–C** depict the backcross derivatives with cultivated okra-type fruits.

### Cross between amphidiploids

Twenty-seven cross derivatives developed by crossing between amphidiploids had also exhibited variation, as revealed by the wide range in characteristics like fruit length (3.30–9.29 cm), number of epicalyx segments (5.00–10.00), number of seeds per fruit (7.33–33.25), petal length (4.90–10.10 cm), petal width (3.77–10.53 cm), and flower diameter (5.70–16.70 cm) (**Table 4C**). B-13 × AM-24, a derivative of cross involving *A. esculentus*, *A. angulosus* var. *grandiflorus*, *A. tetraphyllus*, and *A. pungens* var. *mizoramensis* (**Table 2D**), exhibited maximum values for fruit length (9.29 cm) and number of epicalyx segments (10.0). The minimum values for fruit width (1.73 cm), epicalyx length (1.20 cm), epicalyx width (0.13 cm), petal length (3.77 cm), petal width (3.77 cm), flower diameter (5.70 cm), and the absence of spiny hairs on the fruit surface were also shown by this derivative (**Supplementary Table 1E**). The plants of B-4 **×** AM-24 and B9 **×** AM-25 were vigorous, with profuse branching and fruiting. However, B-9 **×** AM-23 plants were distinct, with comparatively small flowers (diameter 7.50 cm), broad epicalyces, and broadly lobed leaves and without spiny hairs on the plant parts. Thirteen derivatives possessed either red color throughout or red spots or red striations on the stems. Red coloration at the nodal region was also a frequently observed characteristic in many derivatives.

**Figure 10:**
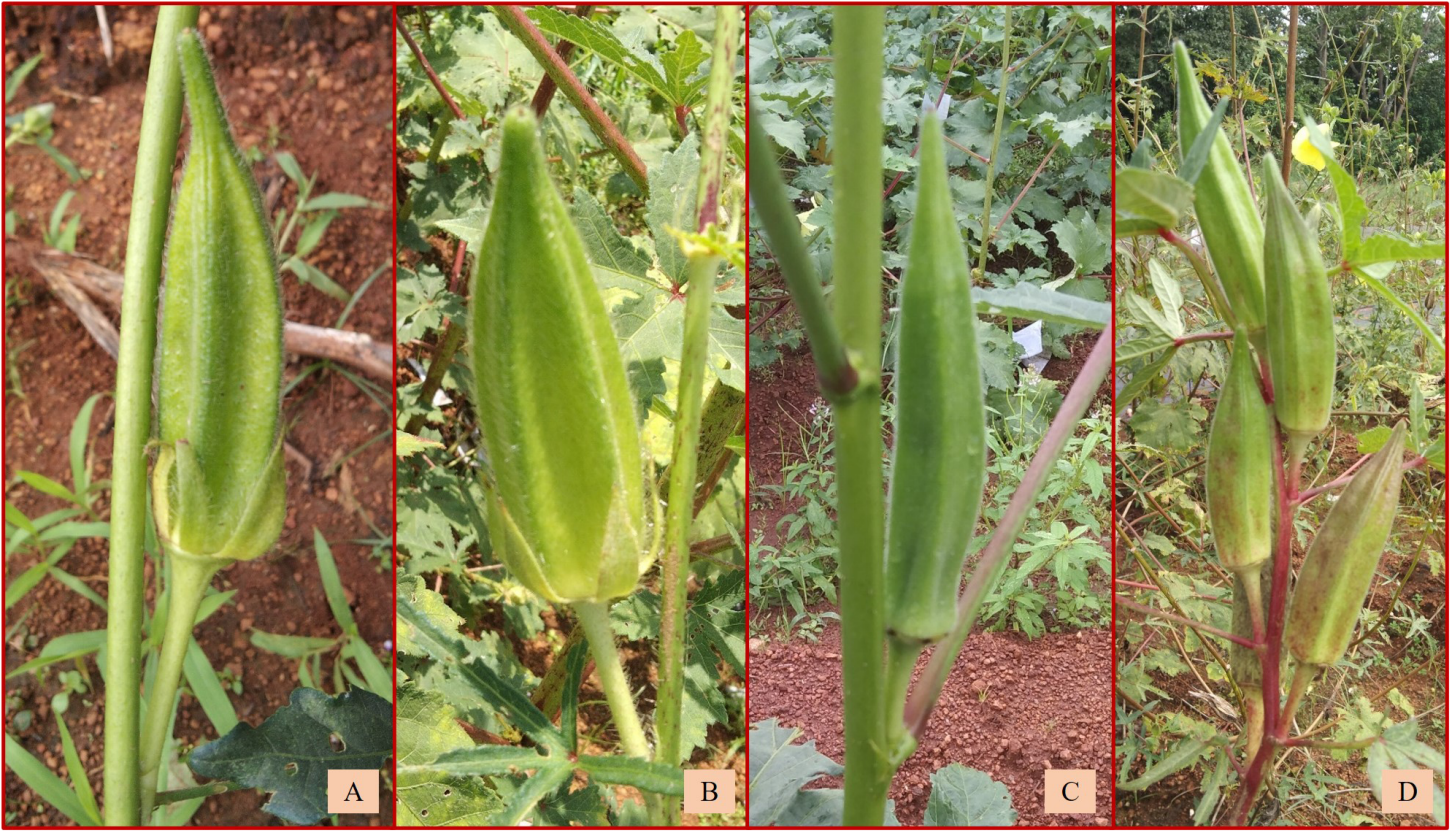
Increase in fruit length in derivatives of cross between amphidiploids **(A)** B-15 **×** AM-25, **(B)** AM-25 **×** CR-1, **(C)** B-13 **×** AM-24, and **(D)** B-16 **×** AM-24.

AM-24 **×** B-4 was a cross between two amphidiploids in which B4 was a progeny of a three-way cross involving Arka Anamika, *A. tetraphyllus* (IC253122), and *A. palianus* (IC0624218). The fruits had an average length of 5.60 cm, a width of 2.70 cm, and six epicalyces measuring 2.76 cm × 0.83 cm, and soft trichomes. The increase in the length of fruits was evident in the derivatives, namely, B-16 **×** AM-24, B-15 **×** AM-25, AM-25 **×** CR-1, and B-13 **×** AM-24 (**Figures 10A–D**). AM-25 × CR-1 (*A. esculentus* IC260106 × *A. sagittifolius* IC470750)]) was the only cross involving *A. sagittifolius* in this category; however, none of the distinct characteristics of *A. sagittifolius* (pink flowers, slender epicalyces, soft fruits, and long peduncles) were observed.

### Multi-cross combinations

All the derivatives of multi-cross combinations had medium-wide epicalyces to wide epicalyces (range 0.43–1.10 cm) with medium spiny hairs on the fruit surface. A wide range was observed in fruit length (3.80–9.70 cm), in contrast to a narrow range in fruit width (2.10–2.55 cm). The number of seeds per fruit exhibited maximum CV (39.48%), and the values ranged from 8.50 (B-9) to 24.33 (B-4). The fruits of B-5 had on average nine epicalyx segments per fruit and fruit length of 9.04 cm. The number of epicalyx segments in the derivatives under this category ranged from 5.0 (B-15) to 9.0 (B-5). However, the fruits of B-15 were short (3.80 cm), with only five epicalyx segments with length and width ranging from 2.90 cm and 0.90 cm, respectively. Fruit peduncle length was maximum (4.08 cm) in B-5 (**Table 4C**; **Supplementary Table 1F**). B-9 exhibited maximum values for flower diameter (14.80 cm); however, B-5 and B-13 exhibited maximum values for petal length and width, respectively.

### Interspecific cross selfed derivatives

**Figure 11:**
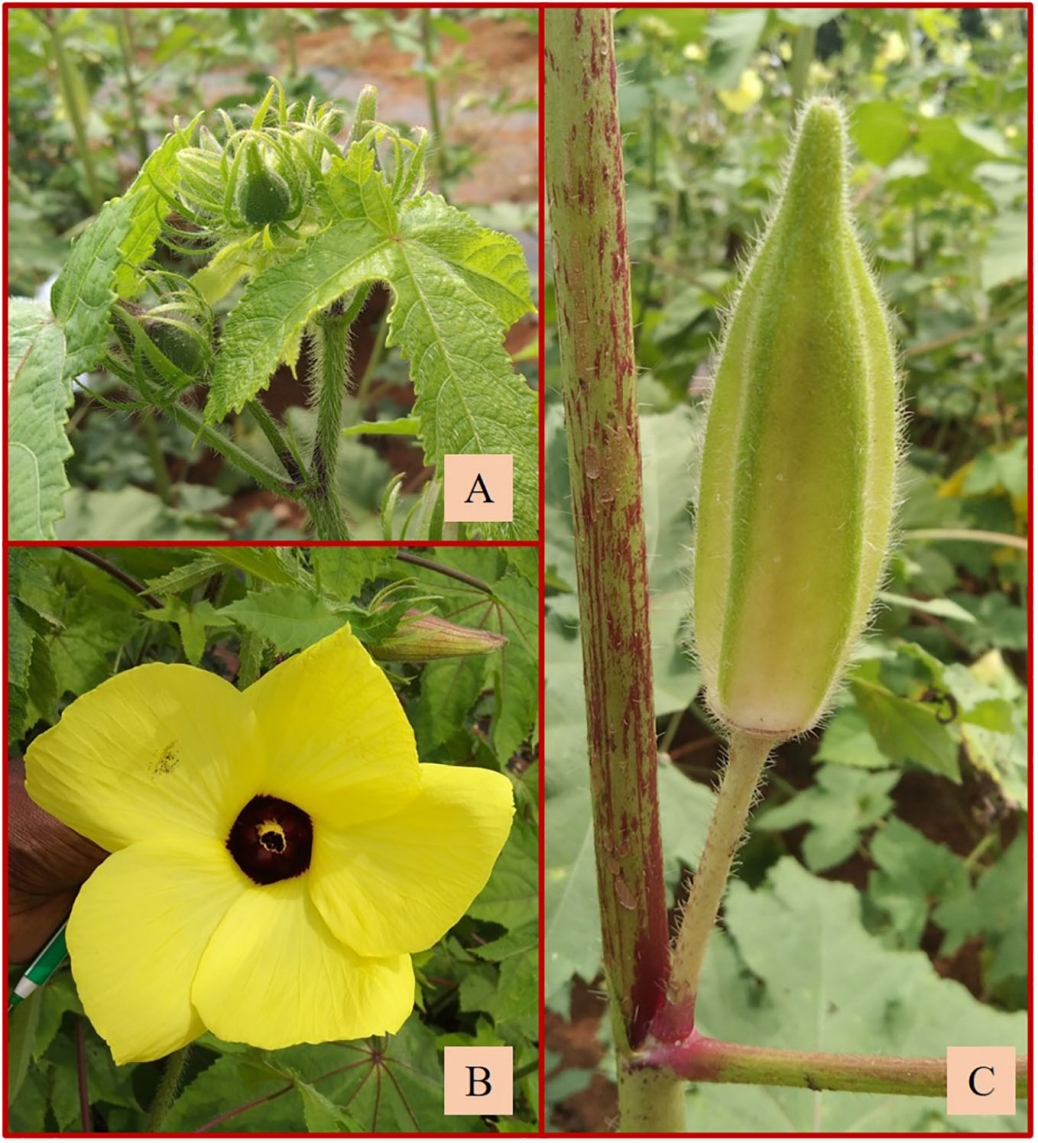
**(A)** Slender epicalyx. **(B)** Bright yellow flower. **(C)** Oblong fruit of AM-19.

Three interspecific cross selfed derivatives of the cross, i.e., *A. sagittifolius* **×** *A. moschatus* subsp. *moschatus*, advanced to the F_8_ generation (**Table 4C**; **Supplementary Table 1G**) were also included in the study. Two of them (AM-13 and AM-19) exhibited the characteristics of *A. moschatus* having fruits with slender linear epicalyces, soft to no hairs, and oblong capsules with acute apex and bright yellow flowers (**Figures 11A–C**). The fruit length and width ranged from 7.82 to 8.56 cm and 2.53 to 2.63 cm, respectively. Similar to *A. moschatus*, the number of epicalyx segments varied from 7.33 to 9.67 with a mean of 8.83 per fruit. The length and width of epicalyx segments were 2.18 cm and 0.38 cm, respectively. In contrast to basal branching, branching throughout the nodes was a peculiar characteristic observed in these derivatives. The flower diameter ranged from 10.77 to 13.05 cm with a mean of 11.74 cm (**Table 4C**). AM-13 did not exhibit spiny hairs on the fruits, and the number of seeds also varied significantly from the other two derivatives (**Supplementary Table 1G**). The fruit peduncle length ranged from 4.30 cm to 5.22 cm with an average of 4.79 cm in the derivatives.

## Discussion

A promising breeding method for the creation of new genetic variability in a crop species is wide hybridization, which became a routine practice in crops such as wheat after the advancement of hybridization techniques (Mujeeb-Kazi and Rajaram, 2002). The dynamics of geneflow existing between the cultivated and wild species of *Abelmoschus* is less studied as compared to other vegetable crops. Earlier studies revealed that “it is more difficult to cross cultivated species with wild species of *Abelmoschus*” as evident from several attempts made for interspecific hybridization by various authors (Joshi and Hardas, 1956; Kuwada, 1957; Hamon and Yapo, 1985). Wild relatives of okra represent a good source of variation for breeding programs, particularly for traits related to biotic and abiotic stresses, and fruit quality (Sandeep et al., 2022). Hence, wild species that remain unexploited may be used via pre-breeding approaches for developing desirable introgressed lines.

In the present investigation, interspecific crosses involving *A. esculentus* and wild relatives, followed by polyploidization, generated considerable variability in the amphidiploid derivatives. These results reaffirmed the earlier reports on the successful interspecific hybridization to generate considerable variability (Reddy, 2010; Arunkumar, 2015; Patel et al., 2021; Badiger et al., 2023; Singh et al., 2023b) and the existence of barriers to the transmission of gene flow from wild species to the cultivated ones (Rajamony et al., 2006; Jatkar et al., 2007; Badiger et al., 2023). Widely variable amphidiploids have also been generated from the hybrids of wheat and related species of the genera *Aegilops*, *Secale*, and *Thinopyrum* by Nemeth et al. (2015). Rapid phenotypic changes in synthetic allopolyploids in *Brassica napus* have also been reported by several workers, which were attributed to mechanisms such as recombination between homoeologous chromosomes and alterations in the expression pattern and DNA methylation status of genes (Song et al., 1995; Schranz and Osborn, 2000; Lukens et al., 2006; Gaeta et al., 2007; Xu et al., 2009; Szadkowski et al., 2010). However, in contrast to this, Sheela (1994) reported a strong genetic mechanism preventing free recombination in the interspecific hybrid population of *Abelmoschus*.

In the present study, the majority of the okra amphidiploids exhibited intermediate morphology, especially with respect to the fruit characteristics, which is in conjunction with the observations in amphidiploids developed between *Cucumis sativus* L. and *Cucumis hystrix* Chakrav. (Chen et al., 2002) and *Solanum melongena* L. and *Solanum aculeatissimum* Jacq. (Zhou et al., 2018), where the synthetic amphidiploid plants possessed some characteristics common either to cultivated or to the wild parents. Further, the greater vigor of the amphidiploids may be attributed to the heterozygosity of different genomes (Nasrallah et al., 2000; Chen, 2010; Fujimoto et al., 2011). Badiger et al. (2023) observed that the interspecific hybrids developed between *A. esculentus* and wild species (*A. manihot* var. *tetraphyllus* and *A. moschatus*) showed intermediate morphological traits with greater alliance toward their wild parents. In the present study, distinct variation was also apparent in the indumentum density among the amphidiploid derivatives. The purple coloration on the stems, petioles, and nodal region in the majority of the amphidiploids was inherited from the wild parent *A. pungens* var. *mizoramensis*. This taxon as described originally by John et al. (2020) exhibits dark-purple blotches at nodes, and these blotches extend to the internodes. It was interesting to note that the recovery and survival rate of the amphidiploids with *A. pungens* var. *mizoramensis* were high compared to those of other combinations (*A. esculentus* with other wild species) after colchiploidization, reaffirming the findings of Rana and Arora (1991) that there is a genetic affinity between *A. esculentus* and *A. pungens*.

### Selfed amphidiploid derivatives

Wild characteristics, namely, shorter fruit length, hispid nature, and seed dehiscence from the apex of the fruit, were prominent in the amphidiploids in the F_1_ generation; however, profuse and extended bearing were observed. As linkage drag was prominent, they required repeated backcrossing to accumulate the desirable genome of the recurrent parent. The similarity in morphology among the C_1_Pusamizo derivatives may be attributed to the heterozygosity in all the F_1_ individuals leading to a uniform homogenous population. The increase in fruit length was evident (range of fruit length 6.13–8.83 cm) (**Table 4A**) against the range of fruit length in *A. pungens* var. *mizoramensis* (3.5–5.0 cm) (**Table 3**). IC624235 and IC624236 (both collections from Nagaland, India) were the *A. pungens* var. *mizoramensis* genotypes used. Among them, a higher seed setting (more than 45 seeds) was obtained in amphidiploids involving IC624235, except in C_1_Pusamizo235-03. This reveals that there is genotype-specific cross compatibility between *A. esculentus* and *A. pungens* var. *mizoramensis*. The persistence of epicalyces at fruit maturity was not observed in the derivatives in contrast to the persistent epicalyces in the wild parent (John et al., 2020). Regarding the derivatives involving *A. esculentus* and *A. moschatus* variant, the seed setting ranged from 22.75 to 98.25, indicating that the seed setting was possible by using *A. esculentus* as a female parent. Badiger et al. (2023) also confirmed that viable seeds were obtained in the direct cross. They reported that *A. moschatus* × *A. esculentus* crosses (reciprocal cross) suffered from severe pre-zygotic barriers that resulted in the formation of aborted embryos with partially filled seeds. C_1_Arkamosc (variant)750-02 produced fruits like *A. moschatus*, confirming the dominance of genes from the wild parent. As *A. moschatus*, a perennial species, is reported to be tolerant to other multiple biotic stresses such as powdery mildew, jassids, whitefly, fruit, and shoot borer (Badiger and Yadav, 2019; Yadav et al., 2019), it can be utilized in multiple pest and disease resistance breeding (Badiger et al., 2023). The perennation tendency and profuse-bearing habit of amphidiploids can also be exploited for selecting segregants to be used as a home garden vegetable, as a single plant in the kitchen garden can suffice the okra requirement of a family throughout the year. For commercial cultivation, genotypes bearing during the off-season will also fetch higher prices for the farmers.

The amphidiploids in the F_2_, F_3_, and F_4_ generations also exhibited wide variations with respect to the number of seeds per fruit. The seed set was even as low as nine seeds per fruit in AM-23, a cross between *A. esculentus* and *A. angulosus* var. *grandiflorus*. This low seed set might be owing to genetic imbalances due to improper bivalent formations during meiosis because of genomic differences in parents (*A. angulosus* var. *grandiflorus* as 2n = 66; *A. esculentus* as 2n = 130) (Merita et al., 2012). The epicalyx width, one of the important taxonomic characteristics, was maximum in the amphidiploid AM-23 (C_3_/87/gran4) (1.60 cm) (**Supplementary Table 1B**), indicating that the trait might have been transferred from the male parent IC613527 (*A. angulosus* var. *grandiflorus*), which has comparable epicalyx width ranging from 1.30 to 1.50 cm in general (**Table 3**) (Yadav et al., 2014). C_2_/Ruchi/tetra1 had plant morphology similar to that of *A. tetraphyllus*, with purplish stems, very vigorous growth, and orthotropic basal branching. It developed fruits with an average length of 7.25 cm and without prickly spines (the only amphidiploid without spiny hairs among the advanced selfed derivatives). This contrasted with the fruit length in *A. tetraphyllus*, which ranges from 3.50 to 5.50 cm (Pandey et al., 2016), indicating the transfer of useful genes from cultivar Ruchi. Similar kinds of naturally introgressed lines (with enhanced fruit length and fusiform shape) can be observed when *A. tetraphyllus* and *A. esculentus* are grown in the adjacent or same fields in the previous season. AM-4, a derivative selected from the cross between *A. esculentus* IC265650 and *A. pungens* var. *mizoramensis* IC0624222, was observed to have a maximum value for flower diameter (13.93 cm), which exceeded the mean flower diameter of both the parents.

All the derivatives produced showed yellow flowers with extended flowering periods, giving scope for identifying segregants with ornamental qualities with perennial nature. We also identified novel types with profuse flowering, suitable for deriving ornamental types in the selections among C_1_Pusamizo14, C_1_Pusamizo02, C_1_Pusamizo30, C_1_Pusamizo09, and C_1_Pusamizo06, which were less hispid too. However, horticultural practices like pruning need to be standardized to maintain them as a bushy plant. Cui et al. (2018) reported the prospects of the development of perennial wheat through hybridization between wheat and wheatgrasses (*Thinopyrum* spp.). Similar valuable trait-specific amphidiploids of *Triticum turgidum* subsp. *durum*–*Aegilops longissima* with high iron and zinc contents (Tiwari et al., 2008) and *T. turgidum*–*Aegilops umbellulata* with strong tillering ability, stripe rust resistance, and seed size-related traits (Song et al., 2019) have also been reported. Similarly in vegetables, the hull-less seed trait was transferred from *Cucurbita pepo* to *Cucurbita moschata* through interspecific hybridization (Kaur et al., 2023).

### Backcrossed derivatives

The traits from wild species exhibit dominance; accordingly, the amphidiploids developed possessed a high hispid nature of the plant parts. To recover the traits of a cultivated parent, backcrossing was initiated in the F_2_/F_3_ stage. Irrespective of the recurrent parent used in backcrossing, our focus was on the selection of segregants with intermediate to long fruits with less spiny hairs on the fruit surface, extended and profuse bearing, and a greater number of seeds per fruit. Wide variability was observed among the BC derivatives with respect to the fruit morphology varying from true wild types to the cultivated types, in addition to the intermediate types. Similar results of morphological divergence among backcrossed progenies developed from the interspecific hybrids of *Brassica oleracea* var. *alboglabra* **×** *Brassica rapa* L. var. *purpurea* were reported by Zhang et al. (2016). The increase in fruit length indicates the gene flow from the cultivated types. Basal branching observed among the derivatives may be a dominant characteristic, as the majority of the derivatives exhibited this characteristic. The segregants without basal branching may be selected in the advanced generations, as this characteristic impedes easy harvesting and reduces the number of plants that can be accommodated in unit areas during commercial cultivation. The characteristic reddish spots and patches along the stems and petioles and tall nature in derivatives with *A. pungens* var. *mizoramensis* as one of the parents indicated that these characteristics might have transferred from this wild parent. *A. pungens* var. *mizoramensis* exhibit purplish spots on the younger stems and attain height up to c. 3 m as reported by Yadav et al. (2014) and John et al. (2020). Similarly, the characteristics, namely, the persistence of epicalyces at fruit maturity and red coloration at the nodal region in C_3_/741/gran12 **×** IC31398A, might have been inherited from the wild parent, *A. angulosus* var. *grandiflorus*, which is reported to have these characteristics (Yadav et al., 2014). C_3_/50/mizo34 **×** IC31340C and C_3_/50/mizo27 **×** IC31340C, the derivatives from the same male parent, had similar morphology. The plants shared characteristics like the presence of reddish spots on the stems, medium-wide epicalyces, and soft to spiny hairs on the stems, leaves, petioles, and fruits. It was observed that seed setting was less than 10 per fruit in 12 backcrossed derivatives (**Supplementary Table 1C**), which may be attributed to the genotype differentiation in crossability behavior.

Among the BC_1_F_3_s, Ruchi **×** AM-24 and Ruchi **×** AM-6 were the desirable segregants with long fusiform fruits with as high as 60 seeds per fruit. Arka Anamika **×** AMPK-1, a derivative in the BC_2_F_3_ generation, also exhibited the morphology of cultivated *A. esculentus* (**Figures 9A–C**). However, more backcrossing will be needed to re-constitute the genome of cultivated okra. The flow of genes from the cultivated to the amphidiploids was apparent in the BC derivative involving Kashi Lalima also. Kashi Lalima is a variety of okra released from ICAR-Indian Institute of Vegetable Research, Varanasi, Uttar Pradesh, India, bearing reddish-purple fruits rich in anthocyanin and phenolics and red-colored stems, petioles, and leaves (**Figure 8D**) (https://iivr.icar.gov.in/kashi-lalima-vror-157 accessed on July 12, 2023). C_3_/50/mizo1 **×** Kashi Lalima exhibited reddish-colored stems, epicalyces, and petioles, which might have been inherited from the cultivated variety. However, it produced green hispid fruits (8.10 × 1.80 cm) with medium broad epicalyces (0.35 cm) inherited from the wild parent (**Figures 8A–D**). The desirable backcrossed derivatives may be screened at hot spot locations of YVMV and ELCV diseases, and the tolerant/resistant lines may be included in the breeding programs. Arunkumar (2015) and Singh et al. (2023b) identified resistant backcross derivatives of the cross between *A. esculentus* and *A. caillei* and *A. esculentus* and *A. manihot*, respectively.

### Crosses between amphidiploids and multi-cross combinations

As a penultimate objective, the amphidiploids were crossed among themselves to induce maximum recombination between the different genomes so that there is a greater probability of obtaining desirable segregants. This was supported by the views of Favero et al. (2020) in *Arachis* sp. that there is a possibility to obtain complex hybrid derivatives generated by crossing between the amphidiploids with high crossability rates. In the present investigation, all the derivatives developed by crossing between amphidiploids yielded plants with prominent basal branching with broader mildly lobed leaves at the bottom and deeply lobed ones at the top. This is in concurrence with the report of Sutar et al. (2013) regarding the presence of angular or palmatifid lower leaves and palmisect upper leaves in *A. palianus*, palmatifid lower leaves in *A. angulosus* Wall. ex Wight et Arn. var. *mahendragiriensis* R.C.Misra var. nov. (Misra et al., 2018), and broadly orbicular lower leaves in *A. pungens* var. *mizoramensis* (John et al., 2020).

B-16 **×** AM-24 was one of the desirable segregants, with medium-long fruits (8.88 cm), closer to the cultivated type, though with some wild-type morphology. The average fruit length of the wild species used in the above cross, viz., *A. tetraphyllus*, *A. angulosus* var. *grandiflorus*, and *A. pungens* var. *mizoramensis*, was 3.50 to 4.50 cm, 3.00 to 4.00 cm, and 3.50 to 5.00 cm, respectively (**Table 3**). Hence, the increase in fruit length can be attributed to the transfer of genes conferring fruit length from the *A. esculentus* genome. This trend also manifested in B-15 **×** AM-25 (8.53 cm), AM-25 **×** CR-1 (8.37 cm), and B-13 **×** AM-24 (9.29 cm). However, this increase was not exhibited in the characteristic number of seeds per fruit, which ranged from 7.33 to 33.25 with an average of 18.50 seeds (**Table 4C**). B-16 **×** AM-23, a derivative of cross involving *A. esculentus*, *A. angulosus* var. *grandiflorus*, and *A. tetraphyllus*, possessed the largest flower among all the derivatives with an average flower diameter of 16.70 cm. Nonetheless, the flower diameter was minimum (5.70 cm) in B-13 **×** AM-24, a smaller value than any of its parents. The flower diameter of the parental species ranged from 6.00 to 7.00 cm in *A. esculentus* and *A. angulosus* var. *grandiflorus*, 7.00 to 8.00 cm in *A. pungens* var. *mizoramensis*, and 5.00 to 7.00 cm in *A. tetraphyllus* (**Table 3**). Except for B-9 **×** AM-25 and B-15 **×** AM-25, all the derivatives had *A. pungens* var. *mizoramensis* as one of their parents. B-9 **×** AM-25 recorded the maximum value for the width of epicalyx segments among the crosses between amphidiploids. Seven derivatives, namely, B-16 **×** AM-24, B-15 **×** AM-25, B-13 **×** AM-24, B-16 **×** AM-23, B-2 **×** AM-25, B-9 **×** AM-23, and B-4 **×** Was-15, were devoid of spiny trichomes on their fruit.

All the multi-cross combinations had medium spiny fruits (**Supplementary Table 1F**). Three derivatives, namely, B-5, B-6, and B-9, had intermediate fruit characteristics, indicating the probability of increasing fruit length through backcrossing. The results are encouraging considering that simultaneous introgression of genes from four wild *Arachis* species into peanuts was possible through the development of complex hybrids by crossing between amphidiploids of *Arachis* spp. (Favero et al., 2020). Further, the derivatives from these complex multi-crosses can be used for conducting basic studies to understand the genetic mechanisms of virus tolerance in *Abelmoschus*. Such derivatives can help in the identification of alternate genes for tolerance in okra.

### Interspecific cross selfed derivatives

In addition to searching germplasm for virus tolerance, interspecific crosses were also made to investigate the prospects of obtaining other desirable traits from the wild germplasm. *A. sagittifolius* (a wild taxon bearing tuberous roots, slender epicalyces, and white to pale yellow to dark pinkish flowers) was crossed with *A. moschatus* subsp. *moschatus* (bearing non-tuberous tap roots, slender epicalyces, and yellow flowers with dark purple-centered corolla). The F_1_s developed yielded plants with deep red color with ornamental potential, both as a candidate for flowerbeds and as potted plants. The ornamental F_1_ hybrids had tuberous tap roots and perennation ability, which can be easily propagated through stem cuttings. They hardly needed any care and could easily grow throughout the year in tropical climates (John et al., 2013a; John et al., 2013b). The F_2_s of the cross produced flowers of various shades of pink, yellow, light red, and dark red and combinations of these colors. However, the selfed derivatives of the present study produced bright yellow flowers only, as yellow-flowered segregants were selected from the early segregating generation. Further, they have attained homozygosity for the characteristics at the F_8_ generation. Both direct and reciprocal crosses can be attempted further to derive more diverse segregants. The derivatives have the potential to act as a source of powdery mildew tolerance in okra breeding.

### Fertility restoration in amphidiploids

The number of seeds set per fruit increased significantly in the amphidiploids, and it ranged from 4.00 to 98.00 per fruit. Seven derivatives produced more than 50 seeds per fruit, which include four selfed amphidiploid derivatives, one backcrossed, and two interspecific cross selfed derivatives. However, the number of seeds was less than 10 in 22 derivatives, which comprised 13 BCs, two selfed, and seven derivatives of the crosses between amphidiploids. The wide range in value for this trait also indicates that there is genotype specificity in crossability between different species, as crosses of wild *Abelmoschus* with different genotypes of *A. esculentus* had different degrees of fertility restoration leading to differed numbers of seeds per fruit (Merita et al., 2012). Profuse fruit bearing was observed in the majority of the amphidiploids even though seed setting propensity was different. Bhat et al. (2014) reported high irregularities in meiotic chromosome pairing such as the formation of multivalent associations, lower frequency of bivalents with more rod over ring bivalents, and higher number of univalents in the interspecific hybrids involving various *Abelmoschus* species. Similar reports of low frequency of multivalent formation and lagging chromosomes were observed during meiosis in synthetic amphidiploids developed from interspecific hybrids between *S. melongena* and *S. aculeatissimum* (Zhou et al., 2018). These results also highlight the need to conduct detailed cytogenetic analyses of interspecific hybrids and their derivatives in *Abelmoschus*. Reports on the chromosome counts of all the available taxa are also limited. **Table 5** depicts the chromosome counts of wild *Abelmoschus* species used in the present study.

**Table 5:** Chromosome numbers of different *Abelmoschus* spp. used in the study.

Taxa	Chromosome number	References
*Abelmoschus moschatus* subsp. *moschatus* and *A. moschatus* subsp*. tuberosus*	2n = 72	Merita et al. (2012)
*Abelmoschus angulosus* var. *grandiflorus*	2n = 66	
*A. angulosus* var. *angulosus*	2n = 56	Ford (1938)
*Abelmoschus caillei* (syn. *Abelmoschus manihot* var. *caillei*)	2n = 194	Singh and Bhatnagar (1975)
	185-199	Siemonsma (1982a); Siemonsma (1982b)
*Abelmoschus esculentus*	2n = 66	Ford (1938)
	2n = 72	Teshima (1933); Ugale et al. (1976); Kamalova (1977)
	2n = 118	Tischler (1931)
	2n = 120	Tischler (1931); Purewal and Randhawa (1947)
	2n = 130	Skovsted (1935); Joshi and Hardas (1953); Joshi and Hardas (1976); Gadwal et al. (1968); Joshi et al. (1974); Singh and Bhatnagar (1975); Merita et al. (2012)
	2n = 132	Medwedewa (1936); Roy and Jha (1958); Breslavetz et al. (1934); Ford (1938)
*A. manihot* subsp. *tetraphyllus*	130	Ugale et al. (1976)
	78	Skovsted (1935)

In general, shorter fruit stalk observed in amphidiploids revealed that the gene controlling this trait has been incorporated from cultivated types. *A. esculentus* is reported to have fruit stalk length ranging from 1.99 to 3.00 cm (Reddy et al., 2022) and that of *A. caillei* ranging from 1.55 to 6.11 cm (AdeOluwa and Kehinde, 2011). However, the fruit stalk length of wild species is longer, viz., *A. palianus* with 4 cm (Sutar et al., 2013), *A. moschatus* with 6.0–15.3 cm (Yadav et al., 2014; Gangopadhyay et al., 2016), and *A. enbeepeegearensis* with 4.3–4.5 cm (John et al., 2013c).

## Conclusion

The interspecific hybridization and subsequent polyploidization created tremendous variability among the amphidiploids. A considerable number of amphidiploids exhibited wild characteristics. However, amphidiploids with cultivated species morphology and intermediate types with longer fruits and less trichome density were also recorded. As the wild relatives used in the wide hybridization are reported to be the source of field resistance/tolerance to okra YVMV and ELCV diseases, the new synthetic amphidiploids would be a valuable source for okra improvement. Out of the 113 amphidiploids characterized, three derivatives, viz., Ruchi **×** AM-24, Ruchi **×** AM-6, and Arka Anamika **×** AMPK-1, expressed the phenotype of the cultivated okra with long fruits, less trichome density and basal branching, and low spininess, which can be directly used as breeding lines. The ornamental potential of interspecific crosses such as AM-13 and AM-19 may also be tapped. Profusely flowering C_1_Pusamizo derivatives could be advanced further for deriving suitable ornamental types. The presence of more seeds per fruit in certain derivatives indicated that fertility restoration and advancing of generation through backcrossing can yield desirable segregants with okra morphology. Other desirable traits like profuse tillering, perennation nature thereby extended flowering and fruiting, off-season bearing, and ornamental qualities can also be exploited. All the derivatives were advanced by backcrossing (72 lines), selfing (48 lines), and bulking the open-pollinated seeds (all 113 lines). Multi-location testing for YVMV disease tolerance of the advanced lines is in progress, which will help to study the reaction of resistance gene(s) in hosts to various strains of YVMV, which in turn will help the breeders to identify major genes controlling known physiological basis of resistance. Hence, the genetic stocks in the form of interspecific derivatives developed here involve seven different wild species and are a reservoir of sources of genes with tolerance to YVMV and ELCV as well as several other characteristics of importance to okra improvement. However, more systematic analyses are required so that genes for tolerance and other traits can be identified and tagged with molecular markers to facilitate their marker-assisted transfer in okra improvement programs. The variations among derivatives for strain level tolerances are to be investigated using this material to enhance their utilization.

The amphidiploid derivatives can also be analyzed through a genome-wide association study (GWAS) so that trait-linked associations can be identified. As reported by Daunay et al. (1993) in eggplant, there is the possibility of bringing back the amphidiploid status to the diploid level, followed by backcrossing with the cultivated okra to easily introgress the valuable traits. Further, there are still chances of evolving diverse derivatives from the bulked population, as segregation was observed in some of the derivatives. As stated by Stalker (2017), the incorporation of genes from the wild species will also contribute to the reduction of production costs, since the introduction of these genes contributes to a decrease in the incidence of diseases, thereby reducing pesticide use and, thus, generating great savings for the producer/farmer.

## Data availability statement

The original contributions presented in the study are included in the article/**Supplementary Material**. Further inquiries can be directed to the corresponding author.

## Author contributions

AS: Data curation, Formal Analysis, Investigation, Writing – original draft, Funding acquisition, Software. JJK: Conceptualization, Data curation, Investigation, Supervision, Writing – review & editing, Resources. KVB: Conceptualization, Methodology, Resources, Validation, Writing – review & editing. ML: Formal Analysis, Investigation, Writing – review & editing. CJL: Data curation, Investigation, Writing – review & editing. MP: Investigation, Resources, Validation, Writing – review & editing. VAMN: Data curation, Formal Analysis, Investigation, Resources, Writing – review & editing. PPT: Data curation, Formal Analysis, Investigation, Writing – review & editing. CP: Resources, Writing – review & editing. SP: Resources, Supervision, Writing – review & editing. AK: Writing – review & editing. RKG: Supervision, Validation, Writing – review & editing. GPS: Project administration, Supervision, Visualization, Writing – review & editing.
