# Supplementary material for: Genetic enhancement of okra [Abelmoschus esculentus (L.) Moench] germplasm through wide hybridization
Source: Front Plant Sci. 2023 Nov 3;14:1284070. doi: 10.3389/fpls.2023.1284070 (PMC10654990; doi:10.3389/fpls.2023.1284070)
Supplement: Supplementary file 1 [file Table_1.docx]

| Supplementary table 1a. Observations on the flower and fruit data of amphidiploids in F_1_ generation | | | | | | | | | | | | | | |
| --- | --- | --- | --- | --- | --- | --- | --- | --- | --- | --- | --- | --- | --- | --- |
| **S. No** | **Amphidiploid name** | **FL (cm)** | **FW (cm)** | **NEpS** | **NR** | **FPL (cm)** | **NSPF** | **EpCL (cm)** | **EpCW (cm)** | **PL (cm)** | **PW (cm)** | **FwD (cm)** | **PSH** |  |
| 1 | C_1_Pusamizo236-29 | 6.87 | 2.13 | 7.00 | 5.00 | 2.13 | 31.00 | 1.85 | 0.80 | 6.60 | 6.90 | 11.45 | 2 |  |
| 2 | C_1_Pusamizo236-26 | 6.13 | 2.40 | 7.00 | 5.00 | 2.67 | 21.00 | 1.98 | 0.58 | 7.40 | 7.97 | 11.43 | 2 |  |
| 3 | C_1_Pusamizo236-25 | 6.53 | 2.33 | 7.33 | 5.00 | 2.57 | 22.00 | 2.03 | 0.87 | 8.07 | 8.87 | 13.00 | 2 |  |
| 4 | C_1_Pusamizo236-24 | 6.63 | 2.23 | 6.00 | 5.00 | 2.47 | 25.00 | 2.10 | 0.83 | 7.73 | 8.37 | 12.33 | 2 |  |
| 5 | C_1_Pusamizo236-22 | 6.23 | 2.23 | 6.67 | 5.00 | 2.33 | 26.00 | 1.80 | 0.73 | 7.13 | 7.90 | 10.47 | 2 |  |
| 6 | C_1_Pusamizo236-21 | 6.42 | 2.43 | 7.33 | 5.00 | 2.23 | 27.00 | 2.27 | 0.73 | 7.73 | 8.47 | 12.73 | 2 |  |
| 7 | C_1_Pusamizo236-18 | 7.70 | 2.93 | 6.67 | 5.00 | 2.10 | 24.00 | 2.33 | 0.83 | 7.93 | 8.80 | 12.27 | 2 |  |
| 8 | C_1_Pusamizo236-16 | 6.53 | 2.53 | 5.33 | 5.00 | 2.33 | 26.00 | 2.13 | 0.97 | 8.37 | 8.40 | 13.17 | 2 |  |
| 9 | C_1_Pusamizo236-15 | 7.20 | 2.43 | 6.33 | 5.00 | 2.57 | 27.33 | 1.73 | 0.60 | 7.47 | 7.00 | 11.57 | 2 |  |
| 10 | C_1_Pusamizo236-20 | 7.33 | 2.23 | 6.67 | 5.00 | 2.47 | 25.67 | 1.87 | 0.60 | 7.40 | 7.23 | 11.43 | 2 |  |
| 11 | C_1_Pusamizo236-27 | 7.43 | 2.10 | 6.00 | 5.00 | 2.57 | 24.33 | 1.70 | 0.60 | 6.97 | 7.23 | 11.20 | 2 |  |
| 12 | C_1_Pusamizo236-05 | 7.23 | 2.33 | 6.00 | 5.00 | 2.64 | 27.33 | 2.13 | 0.63 | 8.20 | 8.33 | 12.33 | 2 |  |
| 13 | C_1_Pusamizo236-06 | 7.33 | 2.43 | 6.00 | 5.00 | 2.54 | 28.33 | 2.03 | 0.60 | 7.77 | 8.57 | 12.33 | 2 |  |
| 14 | C_1_Pusamizo236-07 | 7.43 | 2.23 | 7.33 | 5.00 | 2.46 | 26.33 | 1.63 | 0.60 | 6.70 | 7.23 | 11.10 | 2 |  |
| 15 | C_1_Pusamizo236-11 | 7.53 | 2.10 | 6.33 | 5.00 | 2.60 | 29.67 | 2.83 | 0.50 | 7.23 | 8.57 | 12.17 | 2 |  |
| 16 | C_1_Pusamizo236-12 | 7.33 | 2.33 | 6.33 | 5.00 | 2.57 | 28.67 | 2.03 | 0.93 | 8.27 | 8.90 | 12.73 | 2 |  |
| 17 | C_1_Pusamizo236-14 | 7.13 | 2.23 | 6.67 | 5.00 | 2.67 | 27.33 | 1.50 | 0.47 | 6.87 | 7.37 | 12.33 | 1 |  |
| 18 | C_1_Pusamizo236-02 | 8.47 | 2.03 | 5.33 | 5.00 | 2.77 | 35.00 | 1.93 | 0.83 | 8.23 | 8.87 | 13.73 | 1 |  |
| 19 | C_1_Pusamizo236-30 | 6.80 | 2.13 | 6.67 | 5.00 | 2.27 | 30.00 | 1.60 | 0.47 | 6.80 | 7.73 | 10.87 | 1 |  |
| 20 | C_1_Pusamizo236-04 | 7.23 | 2.10 | 6.33 | 5.00 | 2.47 | 29.67 | 1.53 | 0.40 | 6.63 | 6.73 | 10.87 | 2 |  |
| 21 | C_1_Pusamizo235-09 | 8.68 | 2.58 | 6.33 | 5.00 | 2.52 | 58.00 | 2.17 | 0.63 | 7.67 | 8.27 | 11.20 | 1 |  |
| 22 | C_1_Pusamizo235-04 | 7.20 | 2.65 | 5.00 | 5.00 | 2.43 | 53.50 | 1.87 | 0.87 | 7.73 | 8.20 | 10.90 | 2 |  |
| 23 | C_1_Pusamizo235-03 | 6.25 | 2.40 | 4.33 | 5.00 | 3.35 | 19.50 | 3.17 | 0.90 | 8.13 | 8.40 | 12.07 | 2 |  |
| 24 | C_1_Pusamizo235-02 | 7.36 | 2.70 | 4.67 | 5.00 | 2.84 | 47.75 | 2.13 | 0.93 | 8.50 | 8.57 | 12.03 | 1 |  |
| 25 | C_1_Pusamizo235-01 | 7.30 | 2.90 | 7.00 | 5.00 | 3.24 | 30.75 | 2.47 | 0.67 | 9.57 | 9.77 | 12.77 | 2 |  |
| 26 | C_1_Pusamizo235-06 | 8.18 | 2.65 | 5.67 | 5.00 | 3.16 | 8.50 | 3.07 | 0.83 | 8.07 | 8.80 | 13.23 | 1 |  |
| 27 | C1Arkamosc (variant)750-01 | 7.48 | 2.23 | 8.00 | 5.00 | 3.30 | 22.75 | 2.43 | 0.50 | 8.40 | 7.83 | 12.03 | 2 |  |
| 28 | C1Arkamosc (variant)750-02 | 8.38 | 2.40 | 9.00 | 5.00 | 4.36 | 98.25 | 2.40 | 0.17 | 8.23 | 6.23 | 12.70 | 0 |  |
| 29 | C1Arkamosc (variant)750-04 | 8.83 | 2.63 | 7.33 | 5.00 | 4.48 | 42.50 | 2.23 | 0.63 | 8.37 | 9.30 | 13.13 | 1 |  |
| 30 | C1Arkamosc (variant)750-07 | 8.00 | 2.65 | 7.00 | 5.00 | 2.96 | 35.00 | 2.87 | 0.87 | 9.33 | 9.57 | 12.70 | 2 |  |
| FL - Fruit length; FW - Fruit width; NEpS - No. of epicalyx segments; NR - No. of ridges; FPL - Fruit peduncle length; NSPF - No. of seeds per fruit; EpCL - Epicalyx length; EpCW - Epicalyx width; PL - Petal length; PW - Petal width; FwD - Flower diameter; PSH - Presence of spiny hairs | | | | | | | | | | | | | | |

| Supplementary table 1b. Observations on the flower and fruit characters of amphidiploids in advanced selfed (F_2_, F_3_ and F_4_) generations | | | | | | | | | | | | | | |
| --- | --- | --- | --- | --- | --- | --- | --- | --- | --- | --- | --- | --- | --- | --- |
| **S. No.** | **Amphidiploid code** | **FL (cm)** | **FW (cm)** | **NEpS** | **NR** | **FPL (cm)** | **NSPF** | **EpCL (cm)** | **EpCW (cm)** | **PL (cm)** | **PW (cm)** | **FwD (cm)** | **PSH** |  |
| 1 | AM-2 | 6.04 | 2.38 | 5.33 | 5.00 | 3.24 | 10.60 | 2.37 | 0.90 | 8.37 | 9.17 | 11.37 | 1 |  |
| 2 | AM-3 | 7.70 | 2.70 | 6.67 | 5.00 | 4.14 | 12.50 | 2.70 | 0.73 | 9.30 | 9.43 | 12.93 | 1 |  |
| 3 | AM-4 | 6.24 | 2.56 | 6.67 | 5.00 | 3.36 | 19.40 | 2.47 | 0.70 | 9.53 | 9.80 | 13.93 | 1 |  |
| 4 | AM-5 | 8.72 | 2.63 | 7.00 | 5.00 | 3.52 | 16.00 | 2.37 | 0.70 | 8.33 | 8.50 | 12.33 | 1 |  |
| 5 | AM-6 | 8.78 | 2.40 | 6.00 | 5.00 | 2.32 | 52.75 | 2.07 | 0.77 | 8.67 | 9.67 | 12.57 | 2 |  |
| 6 | AM-8 | 8.96 | 2.65 | 7.67 | 5.00 | 3.56 | 13.50 | 2.80 | 0.77 | 8.30 | 8.47 | 12.33 | 2 |  |
| 7 | AM-9 | 8.42 | 2.30 | 5.67 | 5.00 | 3.40 | 37.75 | 2.27 | 0.63 | 8.80 | 9.23 | 12.47 | 1 |  |
| 8 | AM-10 bulk | 7.82 | 2.53 | 7.00 | 5.00 | 3.62 | 21.25 | 2.83 | 0.90 | 8.60 | 8.10 | 12.77 | 2 |  |
| 9 | AM-12 | 7.16 | 2.28 | 7.00 | 5.00 | 3.06 | 36.25 | 2.93 | 0.90 | 9.53 | 9.50 | 12.63 | 1 |  |
| 10 | AM-14 | 8.18 | 2.48 | 6.67 | 5.00 | 3.78 | 46.25 | 2.90 | 0.73 | 8.67 | 9.23 | 12.20 | 1 |  |
| 11 | AM-15 | 7.90 | 2.35 | 6.67 | 5.00 | 2.78 | 29.40 | 2.33 | 0.80 | 8.37 | 8.57 | 12.50 | 1 |  |
| 12 | AM-17 bulk | 7.44 | 2.30 | 6.00 | 5.00 | 3.24 | 23.75 | 2.60 | 0.63 | 8.23 | 8.00 | 11.20 | 2 |  |
| 13 | AM-23 | 7.20 | 2.80 | 4.00 | 5.00 | 3.80 | 9.00 | 2.80 | 1.60 | 7.27 | 7.33 | 10.30 | 1 |  |
| 14 | AM-24 | 7.62 | 2.75 | 6.00 | 5.00 | 3.34 | 27.25 | 2.53 | 0.87 | 10.00 | 9.37 | 13.53 | 2 |  |
| 15 | AM-25 | 6.23 | 2.67 | 7.67 | 5.00 | 2.78 | 42.33 | 2.53 | 0.77 | 8.70 | 8.17 | 13.43 | 2 |  |
| 16 | C_2_Ruchitetra1 | 7.25 | 1.50 | 7.33 | 5.00 | 2.75 | 25.66 | 2.50 | 0.50 | 7.43 | 7.33 | 10.97 | 0 |  |
| FL - Fruit length; FW - Fruit width; NEpS - No. of epicalyx segments; NR - No. of ridges; FPL - Fruit peduncle length; NSPF - No. of seeds per fruit; EpCL - Epicalyx length; EpCW - Epicalyx width; PL - Petal length; PW - Petal width; FwD - Flower diameter; PSH - Presence of spiny hairs | | | | | | | | | | | | | | |

| Supplementary Table 1c. Observations on fruit and flower characters of 25 back crossed derivatives in BC_1_F_3_ generation | | | | | | | | | | | | | | |
| --- | --- | --- | --- | --- | --- | --- | --- | --- | --- | --- | --- | --- | --- | --- |
| **S. No.** | **Cross** | **FL (cm)** | **FW (cm)** | **NEpS** | **NR** | **FPL (cm)** | **NSPF** | **EpCL (cm)** | **EpCW (cm)** | **PL (cm)** | **PW (cm)** | **FwD (cm)** | **PSH** |  |
| 1 | Ruchi **×** AM-24 (C_2_/50/Mizo34) | 12.55 | 1.60 | 10.00 | 5.00 | 1.55 | 40.50 | 1.40 | 0.10 | 4.30 | 3.60 | 5.90 | 0 |  |
| 2 | Ruchi **×** AM-6 (C_3_/50/Mizo24) | 13.60 | 1.60 | 7.33 | 5.00 | 2.15 | 24.50 | 1.97 | 0.37 | 5.93 | 5.40 | 8.43 | 0 |  |
| 3 | C_3_/50/Mizo34 **×** Salkeerthi | 10.75 | 2.55 | 8.00 | 5.00 | 4.40 | 8.50 | 2.57 | 0.57 | 9.20 | 9.30 | 12.63 | 2 |  |
| 4 | C_3_/50/Mizo27 **×** IC22232 | 8.70 | 2.50 | 6.00 | 5.00 | 3.80 | 9.00 | 2.63 | 0.80 | 7.80 | 8.53 | 11.83 | 0 |  |
| 5 | C_3_/50/Mizo34 **×** Parbhani Kranti | 10.75 | 2.10 | 7.33 | 5.00 | 4.25 | 27.00 | 2.90 | 0.50 | 10.97 | 9.60 | 13.60 | 2 |  |
| 6 | C_3_/741/Mizo9 **×** EC169415 | 9.90 | 2.30 | 7.00 | 5.00 | 2.80 | 26.00 | 2.80 | 0.40 | 7.20 | 8.20 | 11.20 | 1 |  |
| 7 | C_3_/50/Mizo27 **×** Arka Anamika | 10.25 | 2.45 | 7.00 | 5.00 | 3.15 | 5.00 | 2.57 | 0.63 | 7.07 | 7.40 | 11.23 | 1 |  |
| 8 | C_3_/106/Mizo6 (4) **×** IC22232 | 11.20 | 2.30 | 7.00 | 5.00 | 2.90 | 16.00 | 2.95 | 0.65 | 8.40 | 8.30 | 12.65 | 2 |  |
| 9 | C_3_/50/Mizo24 **×** IC31340C | 7.90 | 2.50 | 6.33 | 5.00 | 4.00 | 19.00 | 2.87 | 0.73 | 9.33 | 9.93 | 12.60 | 2 |  |
| 10 | C_3_/50/Mizo27 **×** IC31340C | 8.25 | 2.40 | 8.00 | 5.00 | 3.70 | 4.50 | 2.33 | 0.47 | 7.70 | 8.23 | 9.70 | 1 |  |
| 11 | C_3_/87/gran2 **×** EC169415 | 12.33 | 2.15 | 8.50 | 5.00 | 2.98 | 16.50 | 2.63 | 0.60 | 10.37 | 9.90 | 12.33 | 2 |  |
| 12 | C_3_/741gran12 **×** IC31398A | 10.58 | 2.15 | 7.67 | 5.00 | 3.48 | 19.00 | 2.67 | 0.73 | 8.67 | 9.27 | 13.87 | 1 |  |
| 13 | C_3_/106/Mizo6 (4) **×** Kashi Vibhuti | 7.33 | 2.50 | 8.00 | 5.00 | 3.58 | 28.33 | 2.40 | 0.77 | 9.17 | 9.70 | 14.90 | 0 |  |
| 14 | C_3_/50/Mizo34 **×** South Canara Local | 6.90 | 2.60 | 7.00 | 5.00 | 3.40 | 4.00 | 2.47 | 0.70 | 8.90 | 8.50 | 13.63 | 1 |  |
| 15 | C_3_/106/Mizo6 (2) **×** IC22232 | 10.10 | 2.80 | 6.50 | 5.00 | 4.50 | 6.00 | 2.60 | 0.47 | 8.13 | 8.13 | 10.67 | 2 |  |
| 16 | C_3_/50/Mizo27 **×** IC31398A | 3.80 | 2.20 | 5.00 | 5.00 | 3.10 | 14.00 | 2.67 | 0.54 | 7.03 | 7.67 | 9.23 | 0 |  |
| 17 | C_3_/50/Mizo34 **×** Arka Anamika | 8.38 | 2.80 | 6.33 | 5.00 | 3.64 | 46.75 | 2.43 | 0.70 | 9.63 | 9.67 | 14.87 | 2 |  |
| 18 | C_3_/50/Mizo27 **×** Kashi Vibhuti | 8.08 | 2.60 | 7.00 | 5.00 | 3.93 | 27.00 | 2.37 | 0.77 | 9.27 | 9.23 | 15.17 | 1 |  |
| 19 | C_3_/741/gran12 **×** IC32398A | 5.35 | 2.90 | 7.00 | 5.00 | 3.60 | 9.00 | 2.50 | 0.60 | 6.80 | 6.20 | 9.60 | 2 |  |
| 20 | C_3_/106/Mizo6 (6) **×** Kashi Vibhuti | 9.35 | 2.40 | 7.33 | 5.00 | 3.25 | 6.50 | 3.93 | 0.57 | 9.67 | 9.20 | 14.43 | 1 |  |
| 21 | C_3_/50/Mizo24 **×** South Canara Local | 10.95 | 1.90 | 7.50 | 5.00 | 4.50 | 6.50 | 2.90 | 0.45 | 9.75 | 9.40 | 13.40 | 2 |  |
| 22 | C_3_/50/Mizo1 **×** IC31398A | 7.70 | 2.20 | 6.00 | 5.00 | 3.05 | 7.50 | 2.70 | 0.77 | 7.87 | 8.57 | 13.13 | 1 |  |
| 23 | C_3_/50/mizo1 **×** Kashi Lalima | 8.10 | 1.80 | 8.00 | 5.00 | 3.40 | 10.00 | 2.80 | 0.35 | 8.55 | 8.40 | 10.05 | 1 |  |
| 24 | C_3_/106/mizo6 **×** Hissar Unnat | 9.40 | 2.30 | 7.00 | 5.00 | 3.90 | 6.00 | 3.00 | 0.50 | 9.40 | 9.80 | 12.20 | 2 |  |
| 25 | C_3_/87/gran2 **×** Arka Anamika | 8.53 | 1.95 | 7.33 | 5.00 | 3.05 | 9.53 | 2.43 | 0.43 | 7.63 | 6.93 | 11.83 | 1 |  |
| FL - Fruit length; FW - Fruit width; NEpS - No. of epicalyx segments; NR - No. of ridges; FPL - Fruit peduncle length; NSPF - No. of seeds per fruit; EpCL - Epicalyx length; EpCW - Epicalyx width; PL - Petal length; PW - Petal width; FwD - Flower diameter; PSH - Presence of spiny hairs | | | | | | | | | | | | | | |

| Supplementary table 1d. Observations on fruit and flower characters of five back crossed derivatives in BC_1_F_2_ and BC_2_F_3_ generations | | | | | | | | | | | | | | |
| --- | --- | --- | --- | --- | --- | --- | --- | --- | --- | --- | --- | --- | --- | --- |
| **S. No.** | **Cross** | **FL (cm)** | **FW (cm)** | **NEpS** | **NR** | **FPL (cm)** | **NSPF** | **EpCL (cm)** | **EpCW (cm)** | **PL (cm)** | **PW (cm)** | **FwD (cm)** | **PSH** |  |
|  |  |  |  |  |  |  |  |  |  |  |  |  |  |  |
| 1 | B-13 **×** Arka Anamika | 6.02 | 2.68 | 6.33 | 5.00 | 3.76 | 10.50 | 2.23 | 0.80 | 9.23 | 8.57 | 13.40 | 1 |  |
| 2 | B-16 **×** Arka Anamika | 7.46 | 2.17 | 5.67 | 5.00 | 2.70 | 4.33 | 2.43 | 0.73 | 8.40 | 7.70 | 11.67 | 1 |  |
| 3 | Ruchi **×** AM-25 | 6.43 | 2.57 | 8.00 | 5.00 | 2.68 | 32.33 | 1.80 | 0.80 | 6.90 | 6.60 | 9.50 | 0 |  |
| 4 | Arka Anamika **×** AMPK-1 | 11.40 | 2.00 | 7.00 | 5.00 | 2.60 | 60.00 | 1.90 | 0.90 | 6.80 | 6.50 | 9.30 | 0 |  |
| 5 | Ruchi **×** AMPK-1 | 6.14 | 2.30 | 6.00 | 5.00 | 3.36 | 13.00 | 2.53 | 0.97 | 9.67 | 10.00 | 13.30 | 1 |  |
| FL - Fruit length; FW - Fruit width; NEpS - No. of epicalyx segments; NR - No. of ridges; FPL - Fruit peduncle length; NSPF - No. of seeds per fruit; EpCL - Epicalyx length; EpCW - Epicalyx width; PL - Petal length; PW - Petal width; FwD - Flower diameter; PSH - Presence of spiny hairs | | | | | | | | | | | | | | |

| Supplementary table 1e. Observations on fruit and flower characters of 27 crosses between amphidiploid derivatives | | | | | | | | | | | | | | |
| --- | --- | --- | --- | --- | --- | --- | --- | --- | --- | --- | --- | --- | --- | --- |
| **S. No.** | **Cross** | **FL (cm)** | **FW (cm)** | **NEpS** | **NR** | **FPL (cm)** | **NSPF** | **EpCL (cm)** | **EpCW (cm)** | **PL (cm)** | **PW (cm)** | **FwD (cm)** | **PSH** |  |
| 1 | B-9 **×** AM-24 | 7.30 | 2.80 | 6.67 | 5.00 | 3.46 | 26.75 | 2.63 | 0.87 | 8.93 | 9.20 | 11.07 | 2 |  |
| 2 | B-5 **×** AM-6 | 8.22 | 3.15 | 6.00 | 5.00 | 2.90 | 19.00 | 2.83 | 0.90 | 8.90 | 9.63 | 15.00 | 1 |  |
| 3 | B-2 **×** AM-24 | 8.64 | 2.60 | 7.67 | 5.00 | 3.36 | 33.25 | 2.07 | 0.73 | 7.90 | 8.20 | 12.27 | 1 |  |
| 4 | B-4 **×** AM-24 | 5.60 | 2.37 | 6.00 | 5.00 | 3.85 | 28.33 | 2.60 | 1.00 | 9.03 | 10.07 | 11.43 | 1 |  |
| 5 | B-9 **×** AM-25 | 6.20 | 2.40 | 5.33 | 5.00 | 3.56 | 19.60 | 2.60 | 1.17 | 9.27 | 9.13 | 12.60 | 1 |  |
| 6 | B-16 **×** AM-6 | 5.48 | 2.23 | 6.67 | 5.00 | 3.23 | 10.33 | 2.70 | 0.93 | 8.83 | 10.17 | 14.80 | 2 |  |
| 7 | B-2 **×** AM-6 | 5.90 | 2.40 | 6.33 | 5.00 | 3.22 | 8.75 | 2.47 | 0.73 | 8.93 | 9.17 | 12.93 | 2 |  |
| 8 | B-16 **×** AM-24 | 8.88 | 1.93 | 8.33 | 5.00 | 2.33 | 7.33 | 1.53 | 0.23 | 5.77 | 5.57 | 7.77 | 0 |  |
| 9 | B-15 **×** AM-25 | 8.53 | 1.77 | 6.00 | 5.00 | 2.90 | 33.00 | 2.57 | 0.67 | 9.77 | 9.77 | 13.10 | 0 |  |
| 10 | B-13 **×** AM-6 | 5.76 | 2.70 | 5.67 | 5.00 | 3.72 | 16.00 | 2.67 | 1.13 | 9.17 | 9.93 | 12.33 | 2 |  |
| 11 | B-15 **×** AM-24 | 5.92 | 2.24 | 7.00 | 5.00 | 4.82 | 10.75 | 2.50 | 0.90 | 9.13 | 8.97 | 11.17 | 1 |  |
| 12 | B-9 **×** AM-6 | 5.48 | 2.53 | 5.67 | 5.00 | 3.13 | 19.00 | 2.63 | 1.07 | 9.50 | 9.60 | 11.13 | 2 |  |
| 13 | B-13 **×** AM-24 | 9.29 | 1.73 | 10.00 | 5.00 | 2.42 | 18.00 | 1.20 | 0.13 | 4.90 | 3.77 | 5.70 | 0 |  |
| 14 | AM-24 **×** B-4 | 5.60 | 2.70 | 5.67 | 5.00 | 4.05 | 10.50 | 2.77 | 0.83 | 7.37 | 8.30 | 10.17 | 1 |  |
| 15 | AM-6 **×** (*A. esculentus* x *A. tetraphyllus*)] | 6.14 | 2.70 | 7.00 | 5.00 | 3.00 | 9.75 | 2.97 | 1.07 | 9.90 | 10.33 | 14.30 | 2 |  |
| 16 | B-14 **×** AM-6 | 6.60 | 2.80 | 5.00 | 5.00 | 3.90 | 8.50 | 2.37 | 0.80 | 8.80 | 9.13 | 13.07 | 1 |  |
| 17 | B-13 **×** AM-25 | 5.53 | 2.68 | 6.00 | 5.00 | 4.90 | 30.40 | 2.37 | 0.87 | 9.07 | 8.70 | 14.30 | 1 |  |
| 18 | B-9 **×** AM-10 (bulk) | 6.03 | 2.23 | 6.00 | 5.00 | 3.87 | 18.33 | 2.63 | 0.77 | 9.20 | 9.17 | 13.50 | 1 |  |
| 19 | AM-23 **×** AM-6 | 6.30 | 2.50 | 6.33 | 5.00 | 3.38 | 10.00 | 2.63 | 0.77 | 8.60 | 8.10 | 12.60 | 1 |  |
| 20 | B-12 **×** AM-6 | 5.03 | 1.73 | 5.00 | 5.00 | 1.40 | 22.00 | 2.00 | 0.63 | 6.43 | 5.57 | 7.93 | 1 |  |
| 21 | B-16 **×** AM-23 | 6.70 | 2.20 | 7.00 | 5.00 | 2.20 | 17.00 | 2.30 | 0.90 | 9.60 | 9.50 | 16.70 | 0 |  |
| 22 | B-2 **×** AM-23 | 6.53 | 2.57 | 7.00 | 5.00 | 2.68 | 25.23 | 2.40 | 0.80 | 8.90 | 9.20 | 14.80 | 1 |  |
| 23 | B-2 **×** AM-25 | 4.80 | 2.70 | 7.00 | 5.00 | 3.60 | 9.50 | 2.20 | 0.60 | 7.20 | 7.30 | 8.10 | 0 |  |
| 24 | B-14 **×** AM-25 | 6.10 | 2.57 | 7.00 | 5.00 | 3.63 | 10.33 | 2.70 | 0.83 | 10.10 | 10.53 | 15.60 | 1 |  |
| 25 | B-9 **×** AM-23 | 3.30 | 2.00 | 5.00 | 5.00 | 2.60 | 17.00 | 1.40 | 0.50 | 5.90 | 4.90 | 7.50 | 0 |  |
| 26 | AM-25 **×** CR-1 | 8.37 | 2.23 | 6.00 | 5.00 | 3.03 | 27.67 | 2.65 | 0.60 | 9.20 | 8.20 | 12.95 | 1 |  |
| 27 | B-4 **×** Was15 | 4.90 | 2.40 | 5.67 | 5.00 | 3.50 | 33.00 | 2.67 | 1.07 | 7.80 | 7.90 | 12.17 | 0 |  |
| Gn - Generation; FL - Fruit length; FW - Fruit width; NEpS - No. of epicalyx segments; NR - No. of ridges; FPL - Fruit peduncle length; NSPF - No. of seeds per fruit; EpCL - Epicalyx length; EpCW - Epicalyx width; PL - Petal length; PW - Petal width; FwD - Flower diameter; PSH - Presence of spiny hairs | | | | | | | | | | | | | | |

| Supplementary table 1f. Observations on fruit and flower characters of seven multi-cross combinations | | | | | | | | | | | | | | |
| --- | --- | --- | --- | --- | --- | --- | --- | --- | --- | --- | --- | --- | --- | --- |
| S. No. | Cross | FL (cm) | FW (cm) | NEpS | NR | FPL (cm) | NSPF | EpCL (cm) | EpCW (cm) | PL (cm) | PW (cm) | FwD (cm) | PSH |  |
| 1 | B-16 | 6.60 | 2.50 | 5.67 | 5.00 | 2.95 | 9.00 | 2.67 | 1.10 | 8.37 | 8.97 | 11.30 | 1 |  |
| 2 | B-15 | 3.80 | 2.10 | 5.00 | 5.00 | 3.20 | 12.00 | 2.90 | 0.90 | 6.90 | 7.20 | 9.20 | 1 |  |
| 3 | B-13 | 6.10 | 2.30 | 6.67 | 5.00 | 3.00 | 20.00 | 3.07 | 0.80 | 9.20 | 10.37 | 12.50 | 1 |  |
| 4 | B-9 | 9.70 | 2.45 | 6.67 | 5.00 | 2.80 | 8.50 | 2.57 | 0.47 | 8.97 | 8.50 | 14.80 | 1 |  |
| 5 | B-6 | 9.50 | 2.55 | 7.00 | 5.00 | 3.90 | 22.00 | 2.80 | 0.70 | 8.27 | 7.97 | 11.30 | 1 |  |
| 6 | B-5 | 9.04 | 2.30 | 9.00 | 5.00 | 4.08 | 19.80 | 2.40 | 0.43 | 9.37 | 9.00 | 11.87 | 1 |  |
| 7 | B-4 | 6.53 | 2.17 | 7.33 | 5.00 | 3.97 | 24.33 | 2.57 | 0.57 | 8.37 | 8.97 | 11.33 | 1 |  |
| Gn - Generation; FL - Fruit length; FW - Fruit width; NEpS - No. of epicalyx segments; NR - No. of ridges; FPL - Fruit peduncle length; NSPF - No. of seeds per fruit; EpCL - Epicalyx length; EpCW - Epicalyx width; PL - Petal length; PW - Petal width; FwD - Flower diameter; PSH - Presence of spiny hairs | | | | | | | | | | | | | | |

| Supplementary table 1g. Observations on fruit and flower characters of three inter-specific cross selfed derivatives | | | | | | | | | | | | | | | |
| --- | --- | --- | --- | --- | --- | --- | --- | --- | --- | --- | --- | --- | --- | --- | --- |
| **S. No.** | **Amphidiploid code** | **Gn** | **FL (cm)** | **FW (cm)** | **NEpS** | **NR** | **FPL (cm)** | **NSPF** | **EpCL (cm)** | **EpCW (cm)** | **PL (cm)** | **PW (cm)** | **FwD (cm)** | **PHS** |  |
| 1 | AM-7 | F8 | 8.56 | 2.63 | 7.33 | 5.00 | 4.30 | 11.00 | 2.40 | 0.73 | 8.80 | 8.40 | 11.40 | 2 |  |
| 2 | AM-13 | F8 | 7.82 | 2.53 | 9.67 | 5.00 | 4.84 | 85.25 | 1.73 | 0.17 | 7.43 | 5.70 | 10.77 | 0 |  |
| 3 | AM-19 | F8 | 7.48 | 2.35 | 9.50 | 5.00 | 5.22 | 72.25 | 2.40 | 0.25 | 7.95 | 6.90 | 13.05 | 1 |  |
| FL - Fruit length; FW - Fruit width; NEpS - No. of epicalyx segments; NR - No. of ridges; FPL - Fruit peduncle length; NSPF - No. of seeds per fruit; EpCL - Epicalyx length; EpCW - Epicalyx width; PL - Petal length; PW - Petal width; FwD - Flower diameter; PSH - Presence of spiny hairs | | | | | | | | | | | | | | | |
